# The global impact of *Aspergillus* infection on COPD

**DOI:** 10.1186/s12890-020-01259-8

**Published:** 2020-09-11

**Authors:** Emily E. Hammond, Charles S. McDonald, Jørgen Vestbo, David W. Denning

**Affiliations:** 1 School of Medicine, University of Manchester, Manchester, UK; 2 Division of Infection, Immunity and Respiratory Medicine, Faculty of Biology, Medicine and Health, University of Manchester and Manchester Academic Health Science Centre, Manchester, UK; 3 North West Lung Centre, Wythenshawe Hospital, Manchester University NHS Foundation Trust, Manchester, UK; 4 National Aspergillosis Centre, Education and Research Centre, Wythenshawe Hospital, Manchester University NHS Foundation Trust, Southmoor Road, Manchester, M23 9LT UK

**Keywords:** Fungal infection, Aspergillus, Emphysema, Survival, Incidence

## Abstract

**Background:**

Advanced chronic obstructive pulmonary disease (COPD) often leads to hospitalisation and invasive aspergillosis (IA) is a serious complication. *Aspergillus* sensitisation may worsen symptoms in COPD.

**Methods:**

We identified published papers between January 2000 and May 2019 with > 50 subjects and GOLD criteria for grade II, III or IV (FEV1/FVC < 70% and FEV1 < 80%) using standardised criteria in multiple countries, to re-estimate the prevalence of COPD. Hospitalised COPD patients develop IA in 1.3–3.9%, based on positive cultures of *Aspergillus* spp. and radiological findings. Given limited data on per-patient annual hospitalisation rates, we assumed a conservative 10.5% estimate. Annual IA mortality in COPD was estimated using the literature rates of 43–72%. A separate literature search assessed the impact of *Aspergillus* sensitisation on severity of COPD (by FEV1).

**Results:**

We re-estimated the global prevalence of COPD GOLD stages II-IV at 552,300,599 people (7.39% of the population) with 339,206,893 (8.58%) in Asia, 85,278,783 (8.52%) in the Americas, 64,298,051 (5.37%) in Africa, 59,484,329 (7.77%) in Europe and 4,032,543 (10.86%) in Oceania. An estimated 57,991,563 (10.5%) people with COPD are admitted to hospital annually and of these 753,073 (1.3%) – 2,272,322 (3.9%) develop IA and 540,451–977,082 deaths are predicted annually. *Aspergillus* sensitisation prevalence in COPD was 13.6% (7.0–18.3%) and not related to lower predicted FEV1% (*P* > 0.05).

**Conclusions:**

The prevalence of COPD is much higher than previously estimated. Overall COPD mortality may be higher than estimated and IA probably contributes to many deaths. Improved rapid diagnosis of IA using culture and non-culture based techniques is required in COPD hospital admissions to reduce mortality.

## Background

Chronic obstructive pulmonary disease (COPD) is the most prevalent non-communicable disease of the lungs. It is both preventable and treatable but usually progressive and irreversible [1] with the majority of deaths from COPD occurring in less-developed countries [2]. An ageing population has led to a relative increase in the burden of COPD morbidity and mortality across the world over recent years which has focused the spotlight on COPD as a significant global health concern [2]. The strongest risk factor for the development of airflow limitation is smoking and indoor exposure to biomass [3]; other factors include family history, genetics including alpha-1-antitrypsin deficiency, age, education, tuberculosis, and occupational exposures [4]. Estimates for the prevalence of the disease vary considerably across the globe [5].

Bacteria and viruses are a major cause of COPD exacerbations [6] whereas the role of fungi is less well understood. Most pulmonary fungal infections are caused by members of genus *Aspergillus* [7]. *Aspergilli* are ubiquitous soil-dwelling fungi that have the ability to cause a range of pulmonary conditions including invasive aspergillosis (IA), chronic pulmonary aspergillosis (CPA) including aspergilloma, hypersensitivity pneumonitis, and allergic bronchopulmonary aspergillosis (ABPA) [8]. IA is almost always fatal if not identified and treated early [9]. COPD is one of many underlying risk factors for IA, including neutropenia, liver disease, diabetes mellitus, AIDS, those on immunosuppressive treatment, such as solid and haematopoietic stem cell transplant recipients [9–12]. Prior estimates of the annual global incidence of IA range upwards from 200,000 [9], but difficulties in the precise diagnosis of IA in COPD patients has restrained efforts to include this group in the estimates [10].

One of the aims of this study was to provide global and individual country estimates for the annual number of cases of IA in COPD patients and likely associated mortality. In addition, we wanted to assess whether *Aspergillus* sensitisation contributes to the severity of COPD as recent publications have noted that COPD patients with allergic sensitisation to *Aspergillus fumigatus* experience more symptoms and exacerbations [13], and multiple studies have demonstrated a correlation between asthma severity and sensitisation to *Aspergillus* specifically [14, 15].

## Methods

A systematic review of published literature on PubMed was conducted to ascertain country estimates for 1) COPD prevalence (GOLD grade II-IV), 2) COPD annual hospitalisation rates, and 3) annual cases of IA in COPD patients. Countries included in this review were those with populations over one million, as guided by UN 2015 data [16]. The methodology used in this study is summarised in Additional file 1.

### COPD prevalence data collation

Data were initially collected from three large studies: Burden of Obstructive Lung Disease (BOLD), Proyecto Latinoamericano de Investigacion en Obstruccion Pulmonar (PLATINO) and BREATHE to provide estimates on COPD prevalence, [3, 17, 18]. For countries not included in these studies, a structured literature search and review was conducted to identify other individual country studies to ascertain their estimated prevalence of COPD (Additional file 1). For countries where data was either unavailable or unreliable (see exclusion criteria detailed in Additional file 1), COPD prevalence estimates from neighbouring countries (including countries with BOLD and PLATINO sites) were assumed (Additional file 1).

We pritoritized studies where similar methodologies had been applied at multiple sites. Studies that adopted GOLD criteria were used preferentially; however, a few studies (Rwanda, Estonia and Russia) used lower limit of normal for FEV1/FVC (LLN) to diagnose COPD [19].

Inclusion criteria were:

- Studies published between January 2000 and May 2019;
- Studies with a sample size > 50 people.
- Ssudies with spirometric data were prioritised - use of GOLD criteria to diagnose COPD with spirometry grade II, III or IV (FEV1/FVC < 70% and FEV1 < 80%) or if unavailable then LNN

Exclusion criteria:

- Studies estimating prevalence to be close to or 0%;
- Studies conducted in specific patient groups; e.g., in acutely unwell patients only.

### COPD hospitalisation data collation

Limited data were available for individual countries on their annual rates of COPD-related hospitalisations. The methodology used, similar to that for COPD prevalence data, is summarised in Additional file 1. As many countries did not have published data for hospitalisations, a conservative 10.5% estimate was assumed. This was based on the data from studies in Algeria which have estimated COPD hospitalisation rates at 10.5% [18], not different from the average rate from other studies. The data is therefore more reflective of how many people are admitted, rather than a potentially overestimated figure of how many hospitalisations occur, as it is common for COPD patients to experience more than one exacerbation a year [20].

### Sensitisation systematic review

A systematic review of the prevalence of *Aspergillus* sensitisation in patients with COPD, and the difference in lung function between sensitised and non-sensitised patients was also conducted. The methodological details using the Strengthening the Reporting of Observational Studies in Epidemiology (STROBE) methods [21] are described in Supplementary data. The data extracted were: 1) Authors, publication year, study design 2) Sample size 3) Population characteristics (age/gender) 4) Study location 5) Method of sensitisation assessment 6) Percentage of sample sensitised to *Aspergillus* 7) Lung function of sensitised and non-sensitised patients (FEV1 as a percentage of predicted). The flow-chart in Additional file 2: Supplementary Figure S1 describes the study selection.

Weighted averages and weighted standard deviations were computed to compare lung function between sensitised and non-sensitised patients.

### Invasive aspergillosis data collection

With regards to the incidence of IA in COPD, proportions of the hospitalization estimates from the only four studies are published, from China and Spain, were used to provide an estimated range for each country. The Spanish study estimated 1.3% of COPD hospitalisations to have IA in the last year of their survey, and the later Chinese study estimated 3.9% [10, 20]; the earlier Chinese study estimated 1.9–2.7% [21], in the middle of our range. Their respective mortality rates (71.7 and 43.9% respectively) were also applied to estimate deaths from IA in COPD [10, 20] the earlier study from China had an 80% mortality, but as two patients were excluded from this figure, we elected to use the 71.7% rate as our upper bound of mortality. The most recent study appeared to have a selective admissions policy and was excluded on these grounds [23].

## Results

Overall, COPD prevalence and associated hospitalisation rates, and data for IA prevalence and associated mortality in COPD populations, were estimated for 157 countries globally. Summary estimates are shown in Table 1 and by country in supplementary Table 1 and show significant variability.

**Table 1:** A summary of the data in Table 1, by continent and an overall global estimate of COPD prevalence, hospitalisation rate, IA prevalence in COPD and associated mortality

Region	Population size [16]	COPD population GOLD stages II-IV	COPD annual hospitalisation rate [18]	IA annual rate [10, 20]		IA mortality rate [10, 20]		IA annual incidence per 100,000 population
		n (% of population)	10.5% of COPD population	1.30–3.90% of COPD hospitalisation rate		n (% of IA cases)		
Africa	1,197,415,000	64,298,051 (5.37)	6,751,295	87,767	1.3%	62,929	71.7%	7.3
				271,877	3.9%	115,996	43.0%	22.7
America	1,001,386,000	85,278,783 (8.52)	8,954,272	116,406	1.3%	83,463	71.7%	11.6
				349,217	3.9%	150,163	43.0%	34.9
Asia	4,476,519,000	339,206,893 (8.58)	35,616,724	462,894	1.3%	331,895	71.7%	10.3
				1,389,052	3.9%	597,292	43.0%	31.0
Europe	765,958,000	59,484,329 (7.77)	6,245,855	81,196	1.3%	58,218	71.7%	10.6
				247,744	3.9%	106,530	43.0%	32.3
Oceania	37,131,000	4,032,543 (10.86)	423,417	5504	1.3%	3947	71.7%	14.8
				16,513	3.9%	7101	43.0%	44.5
Globally	7,478,409,000	552,300,599 (7.39)	57,991,563	753,073	1.3%	540,451	71.7%	10.0
				2,272,322	3.9%	977,082	43.0%	30.40

### Prevalence

The global prevalence of COPD GOLD stages II-IV is estimated at 552,300,599 people (7.39% of the population) with 339,206,893 (8.58%) in Asia, 85,278,783 (8.52%) in the Americas, 64,298,051 (5.37%) in Africa, 59,484,329 (7.77%) in Europe and 4,032,543 (10.86%) in Oceania. China and India account for over 82.7 million (5.12% of the Chinese population) and 93.9 million (7.10% of the Indian population) respectively of the global COPD population. Additional file 2 illustrates how the prevalence of COPD varies greatly across the globe. The lowest prevalence of COPD is documented in Peru (1.78%) and only Morocco, Angola, Cameroon, Chad, Republic of Congo, Democratic Republic of Congo, Equatorial Guinea, Gabon, Colombia and United Arab Emirates have estimated COPD prevalence below 3% of the population. The highest prevalences were found in South Africa and the USA by the BOLD study, 19.1 and 14.3% respectively [3], followed by Trinidad and Tobago, Iraq and Denmark (with 14.1, 13.5 and 12.7% respectively). Some of the lowest results were from countries included in the BREATHE study: the United Arab Emirates and Morocco, with 1.9, and 2.2% respectively [18]; in Pakistan COPD prevalence was estimated at 2.1% in the BREATHE study but has since been updated with data from Karachi to 13.8% [24], throwing these very low rates into question.

### Hospitalisation

An estimated 57,991,563 people with COPD are admitted to hospital annually. There are more admissions than this because the mean number of admissions per year for those admitted is often > 1, albeit variable. The rates of admission vary by country, but in most studies it is not clear if these are admission numbers or individuals.

### *Aspergillus* sensitisation

We identified 122 articles via systematic search and an additional three were identified manually (Additional file 2: Supplementary Figure S1). After 27 duplicates were removed, the remaining 98 were screened by title and abstract; of these papers, 86 were excluded and 12 full text articles were assessed for eligibility and 5 were deemed suitable for inclusion [25–29]. These five papers describe 1028 patients with COPD, 720 (70%) male with a mean age of 68.8 years (Table 2). Two of the studies were located in Europe, one in Latin America, one in Southern Asia and one in Eastern Asia.

**Table 2:** Characteristics of included articles on *Aspergillus fumigatus* sensitisation and COPD populations

Author, year	Study Location	Study design	Mean age, years	Gender		Population		Assessment of sensitisation
				Male	Female	Control	COPD	
Jin et al, 2014 [25]	Beijing, China	Cross-sectional	77	174	99	150	273	Plasma IgE analysis
Everaerts et al, 2017 [26]	Leuven, Belgium	Cross-sectional	67	215	85	50	300	Plasma IgE analysis
Agarwal et al, 2010 [27]	Chandigarh, India	Prospective case-control	57	179	21	100	200	Skin prick test
Bafadhel et al, 2014 [28]	Leicester, UK	longitudinal study	69	89	39	22	128	Skin prick test and Plasma IgE analysis
Le Pape et al, 2018 [29]	Bogotá, Colombia	Cross-sectional	74	63	64	-	127	Plasma IgE analysis

We estimate that the total global population of COPD patients who are sensitised to *Aspergillus* is just over 75 million. Serum IgE was the most common method to assess the prevalence of *Aspergillus* sensitisation. The highest prevalence of sensitisation was in Europe based on studies located in Belgium and the United Kingdom (Table 3). In Belgium, Everaerts et al. reported the highest prevalence of 18.3% sensitised to *Aspergillus* [26]. In contrast, Le Pape et al. in Colombia found the lowest prevalence at 7.9% [29]. The weighted average of sensitisation to *Aspergillus* across all studies was 13.6%. The maximum proportion of healthy controls (*n* = 322) sensitised to *Aspergillus* was 4% (Table 4). The FEV1 was 2.5% lower in COPD patients sensitised to *Aspergillus* than non-sensitised patients (*P*-value = 0.57) (Table 4).

**Table 3:** Prevalence of Aspergillus sensitisation in patients with COPD.

Location, author, year	Sensitised to Aspergillus, %		Number of patients
	Control	COPD	
China, Jin et al, 2014 [25]	-	15.0	273
Belgium, Everaerts et al, 2017 [26]	4.0	18.3	300
India, Agarwal et al, 2010 [27]	0.0	8.5	200
United Kingdom, Bafadhel et al, 2014 [28]	-	13.0	128
Colombia, Le Pape et al, 2018 [29]	-	7.9	127
Weighted average:	2.4	13.6	Total: 1028

**Table 4:** Comparison of FEV1 between sensitised and non-sensitised patients with COPD 7.0–18.3% (weighted mean 13.6%

Author, year	FEV1 % of predicted		Number of patients
	Sensitised	Non-sensitised	
Jin et al, 2014 [25]	37	41	142
Everaerts et al, 2017 [26]	41	43	300
Agarwal et al, 2010 [27]	52.9	48.9	200
Bafadhel, 2014 [28]	39	51	128
Weighted average (weighted SD)	43.0 (7.0)	45.5 (4.6)	

### Invasive Aspergillosis

There are only three studies documenting the incidence of IA in those admitted to hospital [10, 20, 22]. All are based primarily on sputum culture positive for *Aspergillus* spp. with supportive radiological findings and usually both a failure to respond to antibiotics and a progressive course. One study included *Aspergillus* antigen and beta 1,3-D-glucan serum biomarkers [22]. These three studies provided the upper and lower bounds of our estimates for IA - 753,073 - 2,272,000 annual cases. As shown in Table 1, the number affected by continent were variable and vary from a low of 14.8/100,000 in Oceania to a high of 34.9/100,000 in the Americas. Annual incidence vary from a low of 1.77 in Panama to 26.07/100,000 in South Africa and countries neighbouring South Africa (Botswana, Eswatini, Lesotho and Namibia) (Additional file 2) using the lower estimate of 1.3% affected. The higher estimate of 3.9% of COPD hospitalisations affected increases the annual incidence to 5.32 to 78.21/100,000.

There are no data published directly estimating the prevalence of CPA complicating COPD, even though about 33% of CPA patients have COPD as an underlying diagnosis, 9% as the primary underlying pulmonary disorder [30]. One recent 12-year retrospective inpatient study from Caruña, Spain which used positive cultures as the enrolment criteria found 68 patients with IA and 7 with CPA [31]. If this ratio pertains in other countries, then CPA complicating COPD would have an inpatient incidence of 76,000 – 272,000. These patients might be included in the overall IA group, as it is likely that this chronic subset of aspergillosis cases was not separated out in the IA COPD studies [10, 20]. Another selective study in China of 36 cases separated them into 25 with IA and 11 with CPA [23]. As most patients with CPA are diagnosed and managed as outpatients, the prevalence of CPA complicating COPD is probably 4-10 times greater than this.

The untreated mortality of invasive aspergillosis is generally thought to be about 100% but is not carefully documented in COPD patients because of the difficulties in establishing the diagnosis early in the course of disease [22]. We took a conservative view of the likely mortality, by using the rates in the two papers where the diagnosis was actively sought and antifungal treatment usually administered, which is probably not the case in most centres. Applying these mortality rates of 44–72%, 540,451–977,082 deaths from IA in hospitalised COPD patients are predicted annually.

## Discussion

There has not been a global COPD prevalence review conducted based on spirometry and standardised assessments for more than a decade, until this year, published as this was paper was being reviewed [32]. The results from our review of the global data estimate there to be 551,792,354 individuals suffering with COPD, the majority of whom are classed as GOLD moderate to severe. Given that the most recent global estimate for COPD prevalence was 251 million [33], now up to 300 million according to the Global Burden of Disease consortium [32], our estimate is much higher even excluding countries with populations under 1 million and excluding GOLD stage I data. Our estimate is dwarfed by another recent systematic review and meta-analysis based on 60 papers and 128,000 patients – a global prevalence of 12.1% (approximately 909 million) [34]. These much higher prevalence figures are not surprising, with under-diagnosis of COPD being one of the major issues facing the management of the disease. During the EPI-SCAN study, Miravitlles et al. found 73% of the patients in their trial with non-reversible airflow obstruction characteristic of COPD did not have a diagnosis before the study [35]. Furthermore, it has been found that this under-diagnosis appears to affect women disproportionately [36]. In order to minimise the effect of under-diagnosis in our estimate we only used internationally accepted spirometry criteria to define COPD, with the exception of a small number of studies using alternative justified methodology. In contrast, our estimate for the global prevalence of COPD at 8.3% is lower than the estimate of 8.9% made in a review by Halbert et al [37]; this is the only review of global COPD prevalence defined by spirometry we could identify. Over 20% of the studies included by Halbert et al. in their analysis defined COPD as GOLD stage I or above (FEV1/FVC < 0.7), this could contribute to their higher estimate of prevalence as we only included GOLD stage II or above in our analysis. We chose to exclude GOLD stage I from our review because using this to define COPD has been shown to over-diagnose elderly patients [38], and there is greater realisation of the variable size of lungs as a result of childhood pollution and other factors interfering with normal lung development [39]. We opted to select studies defining COPD by GOLD stage II-IV over LLN criteria to increase consistency within our review, because the GOLD criteria are more commonly used to define COPD in epidemiology studies, and because using LLN criteria may underdiagnose patients [40].

There are some limitations to the methodology used for calculating prevalence. We did use whole population prevalence, rather than restricting to only those over 40 years of age, as increasing reports of young people with COPD are emerging. As countries with populations less than 1 million were not included, the true total COPD prevalence globally may be slightly higher. The exclusion of GOLD stage I COPD under-estimates mild COPD. We adopted this stance partly because many patients with GOLD stage I COPD (FEV1 ≥ 80%) are undiagnosed [41] and country data are probably unreliable and under-representative. A good example is data from United Arab Emirates, Pakistan and Morocco, with COPD prevalence of 1.9%, 2.1% and 2.2%, respectively [42]. Given the high rates of smoking in these countries [43], we do not consider these prevalence rates to be reflective of their true COPD prevalence. The prevalence used for over half the countries included in our review was based on assumptions taken from other countries. Further data are required, in particular for Sub-Saharan Africa and certain parts of Europe, also recognized by Halbert et al [37]. A more recent systematic review analysing the burden of COPD in Latin America and the Caribbean was only able to identify one paper in the Caribbean region [44].

Biomass fuels and mining are also causative factors in the onset of reduced lung function and progression of COPD [45, 46]. A study in Eastern Europe highlighted a past medical history of pneumonia or tuberculosis (TB) as additional risk factors for the development of COPD [47]. In areas where TB is more prevalent, such as India (which accounted for 24% of total TB cases in 2016 [48]) and South Africa, this could be a causative factor for a higher prevalence of COPD. In 2019, 62% of cases of TB were in South-East Asia and the Western Pacific [48], which could explain why the results of this study showed high prevalence of COPD in Asia.

Life expectancy is another factor influencing COPD prevalence; countries with lower life expectancies, such as in Sub-Saharan Africa [49], may have a lower prevalence for moderate to severe COPD due to patients dying from other causes before their COPD can progress. There is limited published data available for the majority of Sub-Saharan Africa and so it is difficult to highlight a specific country from the results to illustrate this. Further studies are needed to ascertain the prevalence of COPD in this region.

The BREATHE study was one of the large studies used initially to provide COPD prevalence estimates for a number of countries in the Middle East and North Africa. The study used an epidemiological survey to diagnose COPD and estimate its prevalence [18, 42], therefore, unlike the rest of the data included in this review, spirometry was not used as a diagnostic tool. The BREATHE study was included given that it provided data for countries in regions where limited data was available, and could therefore also be used as estimates for other neighbouring countries in Africa and the Middle East. The study provides a sound rationale for using the epidemiological survey that COPD is often not diagnosed until it is clinically apparent and moderately advanced, however patients may still be symptomatic, with an impact on quality of life [11]. Reviewing the prevalence figures, they appear to be very conservative, such as a 1.90% prevalence for the United Arab Emirates, where tobacco use is self-reported in 36% of the male population [50].

### COPD hospitalisation rates

Due to wide range of data available for individual country hospitalisation rates for COPD (Table 5), we applied a conservative figure of 10.5% to each country’s COPD figures. In our systematic review, we sought per patient annual rates, rather than the total number of annual COPD hospitalisations that are probably higher. The BREATHE study [18, 42] and Foo et al [59] documented the incidence of hospitalisations per patient, whereas studies conducted by Zhu et al [45] and Mares et al [63] are unclear whether the figures reflect total number of hospitalisations annually or per patient. We did not interrogate individual country databases. There are several country-specific factors that could feed into hospitalisation rates, including access to healthcare and differences in healthcare systems. In developing countries where resource may be limited or a cost is involved in seeing a healthcare professional, hospitalisation rates will be lower than industrialised countries [48]. Our conservative estimate of over 57 million people admitted to hospital comprises 14% of the global number of 421 million hospital admissions in 2009 [51] and is about 6 times more than influenza as estimated in 2017 [52], likely with some duplication. However, there remains much uncertainty about this number of hospitalisations.

**Table 5:** Hospitalisation data by country collated from our systematic literature review. The BREATHE study [18, 42] and Foo et al [59] documented the incidence of hospitalisations per patient, whereas other studies [45, 63] do not clarify whether the figures reflect total number of hospitalisations annually (a patient can have multiple exacerbations a year requiring admission, dependent on their stage of disease), or per patient. A conservative estimate of 10.5%, based on the BREATHE study’s figure for Algeria was used to calculate average hospitalisation rates for all countries

	Country	Study	Study size	COPD annual hospitalisation rate
Africa	Northern Africa			
	Algeria	BREATHE study, 2012 [18, 42]	3,675	10.50%
	Egypt	BREATHE study, 2012 [18, 42]	9,804	20.30%
	Morocco	BREATHE study, 2012 [18, 42]	3,983	13.00%
	Tunisia	BREATHE study, 2012 [18, 42]	1,952	11.30%
Americas	Latin American and the Caribbean			
	Brazil	Foo et al, 2016 [59]	300	20.00%
	Colombia	Alvarez-Moreno et al, 2018 [60]	Population	13.99%
	Mexico	Foo et al, 2016 [59]	328	14.00%
	Trinidad and Tobago	Gugnani and Denning, 2015 [61]	Population	13.00%
	Northern America			
	United States of America	Foo et al, 2016 [59]	1,001	12.00%
Asia	Eastern Asia			
	China	Zhu et al, 2018 [45]	155,777	20.87%
	Japan	Foo et al, 2016 [59]	300	3.00%
	Korea (Republic of)	Foo et al, 2016 [59]	300	5.00%
	South-Eastern Asia			
	Thailand	Chayakulkeeree et al, 2017 [62]	731,450	7.00%
	Southern Asia			
	Pakistan	BREATHE study, 2012 [18, 42]	3,654	13.00%
	Western Asia			
	Jordan	BREATHE study, 2012 [18, 42]	3,596	20.70%
	Lebanon	BREATHE study, 2012 [18, 42]	3,417	28.80%
	Saudi Arabia	BREATHE study, 2012 [18, 42]	9,675	22.20%
	Syrian Arab Republic	BREATHE study, 2012 [18, 42]	3,402	21.90%
	Turkey	BREATHE study, 2012 [18, 42]	14,911	19.30%
	United Arab Emirates	BREATHE study, 2012 [18, 42]	3,482	40.70%
Europe	Eastern Europe			
	Romania	Mares et al, 2018 [63]	Population	22.07%
	Russian Federation	Foo et al, 2016 [59]	301	14.00%
	Northern Europe			
	United Kingdom and N. Ireland	Foo et al, 2016 [59]	305	15.00%
	Southern Europe			
	Greece	Gamaletsou et al, 2016 [64]	Population	7.70%
	Italy	Foo et al, 2016 [59]	302	6.00%
	Spain	Foo et al, 2016 [59]	303	16.00%
	Western Europe			
	France	Foo et al, 2016 [59]	300	8.00%
	Germany	Foo et al, 2016 [59]	300	9.00%
	Netherlands	Foo et al, 2016 [59]	303	7.00%

### *Aspergillus* sensitisation

Early indicators were that *Aspergillus* sensitization would reduce FEV1 and therefore impact on the severity of COPD, but overall this was not the case - only 2.5% lower and not statistically significant. This contrasts with asthma in adults [53], cystic fibrosis [54] and tuberculosis [55], where *Aspergillus* sensitisation is associated with worse lung function, for mechanistic reasons that are not well exemplified. The subgroup of COPD patients with asthma needs more study however, as antifungal therapy of severe asthma with fungal sensitisation improves symptoms [56]. Further, COPD patients with fungal sensitisation exhibit greater eosinophilia, which could alter therapy [57]. A recent paper links fungal, and especially *Aspergillus*, sensitisation to increased exacerbations of COPD [65].

### Invasive aspergillosis

Due to the limited published data for IA in the context of COPD, it is challenging to provide an accurate estimate on an individual country basis. Assumptions of IA rates in COPD have been made based on the large studies from China and Spain [10, 20]; however, several factors can influence these figures, including the severity of COPD. Studies have shown that infection is more common in the more severe stages, III and IV, of COPD [10, 44, 58]. Therefore, the rates of IA will be influenced by the proportion of the patients with more severe COPD, probably by corticosteroid prescribing practice and possibly by exposure to *Aspergillus*. More studies focused specifically on cases of IA in specific country populations are necessary to build a more accurate picture of the risk associated with COPD and invasive fungal disease. The figures from Spain and China do, however, provide an indication of the overall potential risk of those with COPD and the associated likelihood of contracting a fungal infection. From these three studies, we have estimated the global number of cases of IA in COPD populations to be between 760,017 – 2,272,322. This is substantially higher than the previously estimated 200,000 cases of IA per year (of all causes, not solely COPD) [9]. These data, which need confirmation, are important to raise awareness in healthcare systems to include invasive fungal infection as a differential diagnosis in COPD patients presenting with an exacerbation, and to exclude/treat appropriately and quickly and thence lower attributable mortality, which is otherwise high [9, 10, 20]. The high number if IA cases in COPD may surprise clinicians but should perhaps underscore the need to look for more than the usual infection culprits (i.e. *H. influenzae*) in COPD exacerbations. Often, more intense infection diagnostics and imaging beyond an ordinary chest x-ray will be required, although neither serum assays for galactomannan (*Aspergillus* antigen) nor beta 1,3-D-glucan are satisfactory with positive predictive values of 21.1% and 8.7% respectively although high negative predictive values of >99.5% [22]. *Aspergillus* antibody detection in serum and molecular and culture testing of respiratory samples may be optimal, but needs thorough evaluation [7].

The range of mortality associated with IA in COPD populations was calculated to be between 544,932–976,187 deaths annually. Deaths from COPD ranked 10th among all causes of death in 2015, 4th among countries with a middle Socio-Demographic Index, with an estimated 3,188,000 deaths [58]. Lower respiratory infections were thought to have lead to 2,376,700 deaths. Our estimate of deaths from IA are remarkably high given these estimates and need validation with additional epidemiology studies. In one sense they are conservative, as they assume the diagnosis of IA is made and treatment given. Middle-income countries are hit particularly hard by COPD deaths; therefore improved epidemiological data in these areas would facilitate governments and healthcare organisations making better informed decisions to better reduce premature deaths.

## Conclusion

In conclusion, the global burden of COPD is high, and although variable, likely to be a much larger global health problem than previously realised. Our study highlights the need for healthcare professionals to be aware of the possible risk of IA in their patients, and associated mortality, to identify and treat patients sooner, lowering mortality. We hope that this report will encourage further research into COPD epidemiology and the relationship between *Aspergillus* infection and COPD.

## Supplementary information

**Additional file 1.** The methodology tree describing the structured literature searches and process of data collation for country COPD prevalence estimates and COPD hospitalisation rates.

**Additional file 2: Supplementary Figure S1.** Literature searching for articles on *Aspergillus* sensitisation and COPD. **Supplementary Table 1.** Population in thousands; COPD prevalence (GOLD stages II-IV) estimates; number of COPD hospitalisations annually by country; IA prevalence in COPD populations and associated mortality risk. Two data values are given for IA and their respective mortalities, to create a range of minimum and maximum prevalence and mortality with the Spain (1.3% prevalence and respective mortality of 71.7%) and China (3.9% and respective mortality of 43.0%). Mortality is calculated from the figures provided by Spain (71.7%, from the 1.3% prevalence figure calculated) and China studies (43.0%, from the 3.9% prevalence figure calculated).

**Additional file 3.** Estimated global incidence of invasive aspergillosis per 100,000 population, assuming the lower estimate of 1.3% of hospitalised COPD patients. Countries with populations < 1 million were not estimated and are in white.

## Data Availability

All data generated or analysed during this study are included in this published article [and its supplementary information files].

## Abbreviations

ABPA: Allergic bronchopulmonary aspergillosis
COPD: Chronic obstructive pulmonary disease
CPA: Chronic pulmonary aspergillosis
FEV1: Forced expiratory volume in 1 s
FVC: Forced vital capacity
GOLD: Global Initiative for Chronic Obstructive Lung Disease
IA: Invasive aspergillosis
LLN: Lower limit of normal
PLATINO: Proyecto Latinoamericano de Investigacion en Obstruccion Pulmonar
STROBE: Strengthening the Reporting of Observational Studies in Epidemiology
UN: United Nations
USA: United States of America
