# Supplementary figures and images for: The global impact of Aspergillus infection on COPD
Source: BMC Pulm Med. 2020 Sep 11;20:241. doi: 10.1186/s12890-020-01259-8 (PMC7488557; doi:10.1186/s12890-020-01259-8)

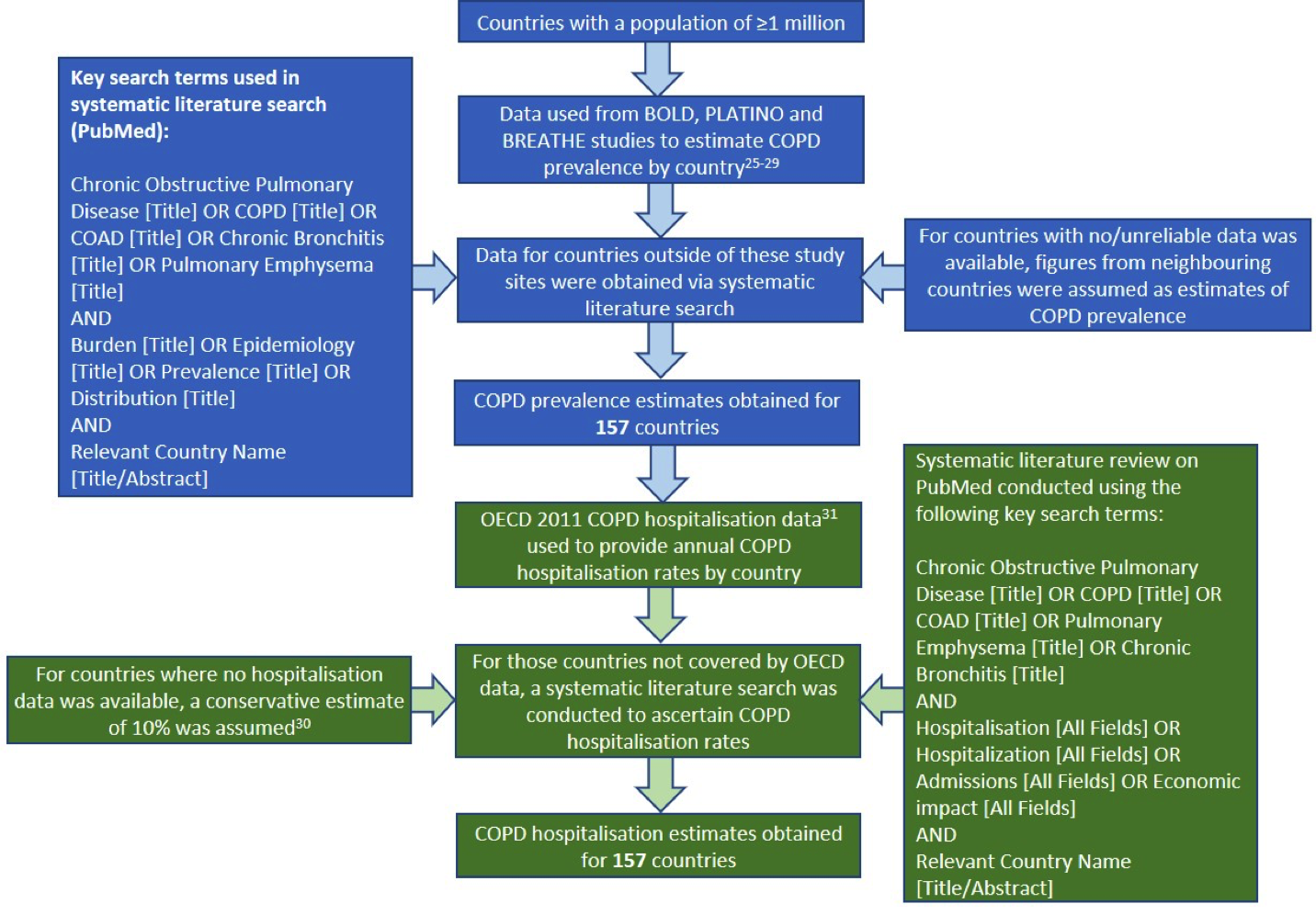

Supplement: Supplementary file 1 — Additional file 1. The methodology tree describing the structured literature searches and process of data collation for country COPD prevalence estimates and COPD hospitalisation rates. [file 12890_2020_1259_MOESM1_ESM.png]

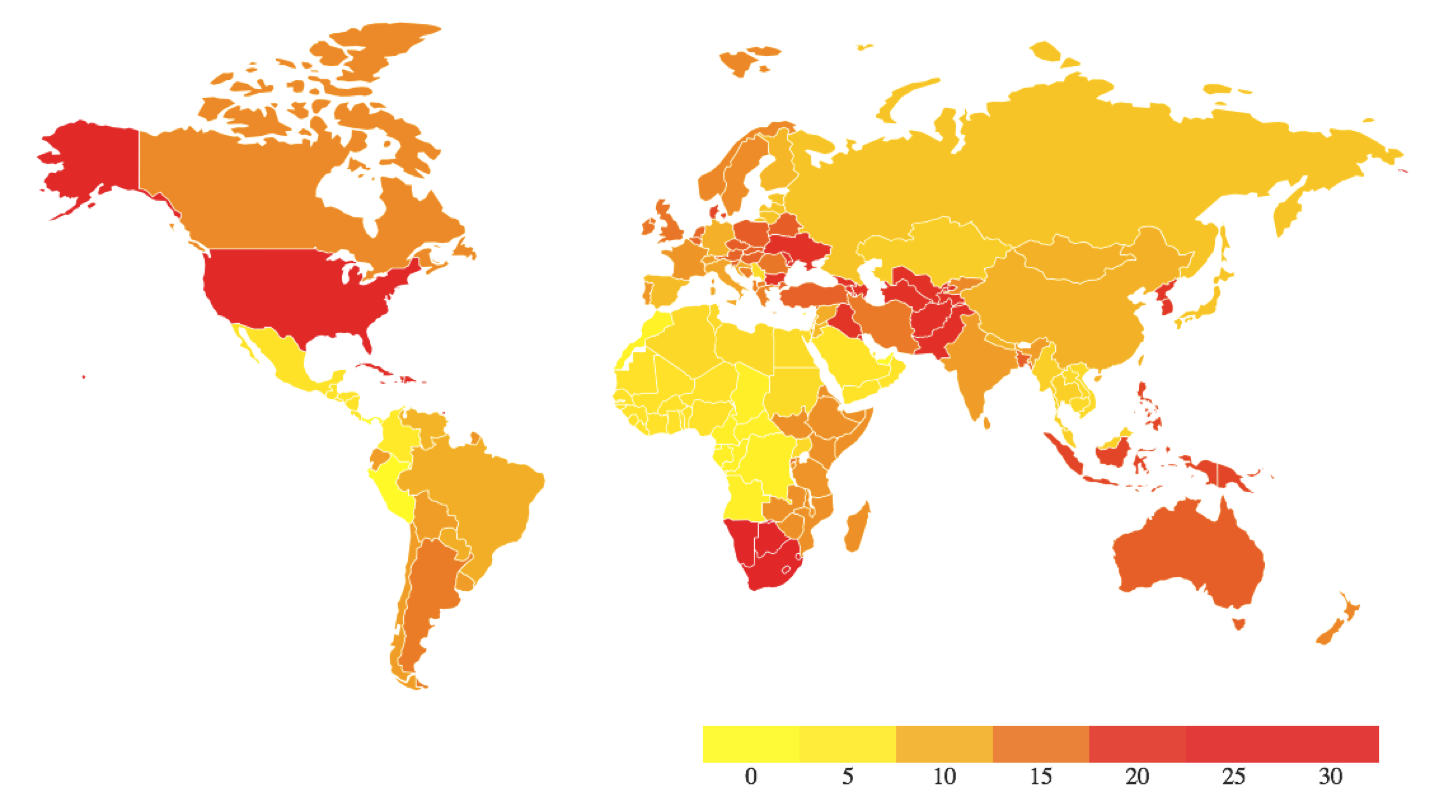

Supplement: Supplementary file 3 — Additional file 3. Estimated global incidence of invasive aspergillosis per 100,000 population, assuming the lower estimate of 1.3% of hospitalised COPD patients. Countries with populations < 1 million were not estimated and are in white. [file 12890_2020_1259_MOESM3_ESM.png]
