# Supplementary material for: The global impact of Aspergillus infection on COPD
Source: BMC Pulm Med. 2020 Sep 11;20:241. doi: 10.1186/s12890-020-01259-8 (PMC7488557; doi:10.1186/s12890-020-01259-8)
Supplement: Supplementary file 2 — Additional file 2: Supplementary Figure S1. Literature searching for articles on Aspergillus sensitisation and COPD. Supplementary Table 1. Population in thousands; COPD prevalence (GOLD stages II-IV) estimates; number of COPD hospitalisations annually by country; IA prevalence in COPD populations and associated mortality risk. Two data values are given for IA and their respective mortalities, to create a range of minimum and maximum prevalence and mortality with the Spain (1.3% prevalence and respective mortality of 71.7%) and China (3.9% and respective mortality of 43.0%). Mortality is calculated from the figures provided by Spain (71.7%, from the 1.3% prevalence figure calculated) and China studies (43.0%, from the 3.9% prevalence figure calculated). [file 12890_2020_1259_MOESM2_ESM.docx]

**Hammond et al, Supplementary data**

Supplemental methods

***Aspergillus* sensitisation and COPD - Systematic Review**

The systematic review we performed aimed to establish the prevalence of Aspergillus sensitisation in patients with COPD, and the difference in lung function between sensitised and non-sensitised patients. The review was performed in accordance with the Preferred Reporting Items for Systematic Review and Meta-Analyses (PRISMA) statement (1). The search was carried out without language restrictions between April 1999 and April 2019 using the following databases: PubMed/Medline, EMBASE, The Cochrane Central and LILACS. The search strategy used for PubMed/MEDLINE simplified is Prevalence OR Epidemiology AND aspergill* AND sensiti?ation AND Chronic Obstructive Pulmonary Disease OR COPD OR COAD OR Pulmonary Emphysema OR Chronic Obstruct* OR Chronic Bronchitis. This automatic search was enhanced by a manual search to identify relevant literature.

We included Cohort studies, randomised control trials, quasi-randomised control trials, cross-sectional studies, case-control studies and case-series studies; they had to have included at least 50 COPD patients. In addition, sensitisation must have been assessed using either serum IgE analysis or skin prick testing. Two reviewers first screened the titles and abstracts of retrieved articles, and subsequently independently assessed the full text of each article against the inclusion criteria. A senior reviewer was available if any disagreements were found. Finally, the remaining studies were analysed for bias using the checklist of essential items laid out by the Strengthening the Reporting of Observational Studies in Epidemiology (STROBE) statement (2). The data extracted were: 1) Authors, publication year, study design 2) Sample size 3) Population characteristics (age/gender) 4) Study location 5) Method of sensitisation assessment 6) Percentage of sample sensitised to *Aspergillus* 7) Lung function of sensitised and non-sensitised patients (FEV_1_ as a percentage of predicted). The flow-chart in Figure S1 describes the study selection.

In order to create global estimates for the prevalence of *Aspergillus* sensitisation from a limited body of literature we used the following system: firstly, the countries where the studies identified were located had this data applied to them. Then, data obtained from China was applied to Central and Eastern Asian countries, excluding Russia. Data obtained from India was applied to Southern and South-East Asian countries, and Papua New Guinea. Data obtained from Colombia was applied to Latin American and Caribbean countries. We created a weighted average from the two European studies we found and applied this data to countries in Europe, North America and Australasia. For the remaining countries in Africa and the Eastern Mediterranean region, we created a weighted average for the prevalence of *Aspergillus* sensitisation from all of the data we obtained in our systematic review and applied it to these areas. Countries were allocated into regions using a system based on the combination of the World Health Organisation regions (3) and the smaller geographic regions presented by the United Nations Statistical Division (4).

Microsoft Excel was used to create the weighted averages for applying sensitisation data globally, and for creating weighted averages and weighted standard deviations for comparing lung function between sensitised and non-sensitised patients.

References

1. Moher D, Liberati A, Tetzlaff J, Altman DG. Preferred Reporting Items for Systematic Reviews and Meta-Analyses: The PRISMA Statement. *Ann Intern Med*. 2009;151(4):264. doi:10.7326/0003-4819-151-4-200908180-00135
2. von Elm E, Altman DG, Egger M, Pocock SJ, Gøtzsche PC, Vandenbroucke JP. The Strengthening the Reporting of Observational Studies in Epidemiology (STROBE) statement: guidelines for reporting observational studies. *J Clin Epidemiol*. 2008;61(4):344-349. doi:10.1016/J.JCLINEPI.2007.11.008
3. World Health Organisation. World Health Statistics 2018. <https://www.who.int/gho/publications/world_health_statistics/en/>. Published 2018. Accessed June 3, 2019.
4. UNSD — Methodology, Standard country or area codes for statistical use (M49). <https://unstats.un.org/unsd/methodology/m49/>. Accessed May 30, 2019.

**Supplementary Figure S1**. *Literature searching for articles on Aspergillus sensitisation and COPD.*

Records identified through database searching
(n = 122)

Screening

Included

Eligibility

Identification

Additional records identified through other sources
(n = 3)

Records after duplicates removed
(n = 98)

Records screened
(n = 98)

Records excluded
(n = 86)

Full-text articles assessed for eligibility
(n = 12)

Full-text articles excluded
(n = 7)

- Abstract only n=3
- Population not representative n=2
- No *Aspergillus* data n=2

Studies included in quantitative synthesis
(n = 5)

**Supplementary Table 1.** *Population in thousands; COPD prevalence (GOLD stages II-IV) estimates; number of COPD hospitalisations annually by country; IA prevalence in COPD populations and associated mortality risk. Two data values are given for IA and their respective mortalities, to create a range of minimum and maximum prevalence and mortality with the Spain (1.3% prevalence and respective mortality of 71.7%) and China (3.9% and respective mortality of 43.0%). Mortality is calculated from the figures provided by Spain (71.7%, from the 1.3% prevalence figure calculated) and China studies (43.0%, from the 3.9% prevalence figure calculated) [3,4].*

|  | **Country** | **Population size (1)** | **COPD population GOLD stages II-IV** | **COPD annual hospitalisation rate (2,3)** | **IA annual rate (3,4)** | | **IA mortality rate(3,4)** | | **Additional information** | |
| --- | --- | --- | --- | --- | --- | --- | --- | --- | --- | --- |
|  |  |  | n (% of population) | 10.5% of COPD population | 1.30 - 3.90% of COPD hospitalisation rate | | n (% of IA cases) | |  |  |
| **Africa** | **Northern Africa** | | | | | | | | | |
|  | Algeria (2,5) | 40,339,000 | 1,492,543 | 156,717 | 2,037 | 1.30% | 1,461 | 71.70% | BREATHE study; | |
|  |  |  | (3.70%) |  | 6,112 | 3.90% | 2,628 | 43.00% | Epidemiological survey | |
|  | Egypt (2,5) | 94,587,000 | 3,310,545 | 347,607 | 4,519 | 1.30% | 3,240 | 71.70% | BREATHE study; | |
|  |  |  | (3.50%) |  | 13,557 | 3.90% | 5,829 | 43.00% | Epidemiological survey | |
|  | Libya (2,5) | 6,288,000 | 232,656 | 24,429 | 318 | 1.30% | 228 | 71.70% | BREATHE study – Algeria data used to estimate COPD prevalence; | |
|  |  |  | (3.70%) |  | 953 | 3.90% | 410 | 43.00% | Epidemiological survey | |
|  | Morocco (2,5) | 35,272,000 | 775,984 | 81,478 | 1,059 | 1.30% | 759 | 71.70% | BREATHE study; | |
|  |  |  | (2.20%) |  | 3,178 | 3.90% | 455 | 43.00% | Epidemiological survey | |
|  | Sudan (2,5) | 38,879,000 | 1,360,765 | 142,880 | 1,857 | 1.30% | 1,332 | 71.70% | BREATHE study – Egypt data used to estimate COPD prevalence; | |
|  |  |  | (3.50%) |  | 5,572 | 3.90% | 2,396 | 43.00% | Epidemiological survey | |
|  | Tunisia (2,5) | 11,452,000 | 423,724 | 44,491 | 578 | 1.30% | 415 | 71.70% | BREATHE study; | |
|  |  |  | (3.70%) |  | 1,735 | 3.90% | 746 | 43.00% | Epidemiological survey | |
|  | **Sub-Saharan Africa** | | | | | | | | | |
|  | Angola (6) | 27,975,000 | 671,400 | 70,497 | 916 | 1.30% | 657 | 71.70% | Cameroon data used to estimate COPD prevalence; | |
|  |  |  | (2.40%) |  | 2,749 | 3.90% | 1,182 | 43.00% | GOLD classification (breakdown of stages not specified) | |
|  | Benin (2,5,6) | 10,635,000 | 340,320 | 35,734 | 465 | 1.30% | 333 | 71.70% | Average of Cameroon and Algeria data used to estimate COPD prevalence; BREATHE; | |
|  |  |  | (3.20%) |  | 1,394 | 3.90% | 599 | 43.00% | GOLD classification (breakdown of stages not specified) | |
|  | Botswana (7,8) | 2,224,000 | 424,784 | 44,602 | 580 | 1.30% | 416 | 71.70% | South Africa data used to estimate COPD prevalence; | |
|  |  |  | (19.10%) |  | 1,739 | 3.90% | 748 | 43.00% | BOLD study; GOLD stage II-IV | |
|  | Burkina Faso (2,5,6) | 18,184,000 | 581,888 | 61,098 | 794 | 1.30% | 569 | 71.70% | Average of Cameroon and Algeria data used to estimate COPD prevalence; BREATHE; | |
|  |  |  | (3.20%) |  | 2,383 | 3.90% | 1,025 | 43.00% | GOLD classification (breakdown of stages not specified) | |
|  | Burundi (6,9-11) | 10,246,000 | 799,188 | 83,915 | 1,091 | 1.30% | 782 | 71.70% | A weighted average from Eastern Africa data used to estimate COPD prevalence; | |
|  |  |  | (7.80%) |  | 3,273 | 3.90% | 1,407 | 43.00% | GOLD stages II-IV | |
|  | Cameroon (6) | 22,967,000 | 551,208 | 57,877 | 752 | 1.30% | 539 | 71.70% | Cameroon data used to estimate COPD prevalence; | |
|  |  |  | (2.40%) |  | 2,257 | 3.90% | 971 | 43.00% | GOLD (breakdown of stages not specified) | |
|  | Central African Republic (6) | 4,577,000 | 109,848 | 11,534 | 150 | 1.30% | 108 | 71.70% | Cameroon data used to estimate COPD prevalence | |
|  |  |  | (2.40%) |  | 450 | 3.90% | 193 | 43.00% | GOLD (breakdown of stages not specified) | |
|  | Chad (6) | 14,071,000 | 337,704 | 35,459 | 461 | 1.30% | 331 | 71.70% | Cameroon data used to estimate COPD prevalence; | |
|  |  |  | (2.40%) |  | 1,383 | 3.90% | 595 | 43.00% | GOLD (breakdown of stages not specified) | |
|  | Congo (6) | 5,025,000 | 120,600 | 12,663 | 165 | 1.30% | 118 | 71.70% | Cameroon data used to estimate COPD prevalence; | |
|  |  |  | (2.40%) |  | 494 | 3.90% | 212 | 43.00% | GOLD (breakdown of stages not specified) | |
|  | Congo (6) (Democratic Republic of the) | 76,590,000 | 1,838,160 | 193,007 | 2,509 | 1.30% | 1,799 | 71.70% | Cameroon data used to estimate COPD prevalence; | |
|  |  |  | (2.40%) |  | 7,527 | 3.90% | 3,237 | 43.00% | GOLD (breakdown of stages not specified)) | |
|  | Côte d'Ivoire (2,5,6) | 23,220,000 | 743,040 | 78,019 | 1,014 | 1.30% | 727 | 71.70% | Average of Cameroon and Algeria data used to estimate COPD prevalence; BREATHE; | |
|  |  |  | (3.20%) |  | 3,043 | 3.90% | 1,308 | 43.00% | GOLD classification (breakdown of stages not specified) | |
|  | Equatorial Guinea (6) | 1,179,000 | 28,296 | 2,971 | 39 | 1.30% | 28 | 71.70% | Cameroon data used to estimate COPD prevalence; | |
|  |  |  | (2.40%) |  | 116 | 3.90% | 50 | 43.00% | GOLD (breakdown of stages not specified) | |
|  | Eritrea (6,9-11) | 4,876,000 | 380,328 | 39,934 | 519 | 1.30% | 372 | 71.70% | A weighted average from Eastern Africa data used to estimate COPD prevalence; | |
|  |  |  | (7.80%) |  | 1,557 | 3.90% | 670 | 43.00% | GOLD stages II-IV | |
|  | Eswatini (7,8) | 1,327,000 | 253457 | 26,613 | 346 | 1.30% | 248 | 71.70% | South Africa data used to estimate COPD prevalence; | |
|  |  |  | (19.10%) |  | 1,038 | 3.90% | 446 | 43.00% | BOLD study; GOLD stage II-IV | |
|  | Ethiopia (6,9-11) | 100,469,000 | 7,836,582 | 822,841 | 10,697 | 1.30% | 7,670 | 71.70% | A weighted average from Eastern Africa data used to estimate COPD prevalence; | |
|  |  |  | (7.80%) |  | 32,091 | 3.90% | 13,799 | 43.00% | GOLD stages II-IV | |
|  | Gabon (6) | 1,950,000 | 46,800 | 4,914 | 64 | 1.30% | 46 | 71.70% | Cameroon data used to estimate COPD prevalence; | |
|  |  |  | (2.40%) |  | 192 | 3.90% | 82 | 43.00% | GOLD (breakdown of stages not specified) | |
|  | Gambia (2,5,6) | 1,986,000 | 63,552 | 6,673 | 87 | 1.30% | 62 | 71.70% | Average of Cameroon and Algeria data used to estimate COPD prevalence; BREATHE; | |
|  |  |  | (3.20%) |  | 260 | 3.90% | 112 | 43.00% | GOLD classification (breakdown of stages not specified) | |
|  | Ghana (2,5,6) | 27,746,000 | 887,872 | 93,227 | 1,212 | 1.30% | 869 | 71.70% | Average of Cameroon and Algeria data used to estimate COPD prevalence; BREATHE; | |
|  |  |  | (3.20%) |  | 3,636 | 3.90% | 1,563 | 43.00% | GOLD classification (breakdown of stages not specified) | |
|  | Guinea (2,5,6) | 12,152,000 | 388,864 | 40,831 | 531 | 1.30% | 381 | 71.70% | Average of Cameroon and Algeria data used to estimate COPD prevalence; BREATHE; | |
|  |  |  | (3.20%) |  | 1,592 | 3.90% | 685 | 43.00% | GOLD classification (breakdown of stages not specified) | |
|  | Guinea-Bissau (2,5,6) | 1,777,000 | 56,864 | 5,971 | 78 | 1.30% | 56 | 71.70% | Average of Cameroon and Algeria data used to estimate COPD prevalence; BREATHE; | |
|  |  |  | (3.20%) |  | 233 | 3.90% | 100 | 43.00% | GOLD classification (breakdown of stages not specified) | |
|  | Kenya (6,9-11) | 47,456,000 | 3,701,568 | 388,665 | 5,053 | 1.30% | 3,623 | 71.70% | A weighted average from Eastern Africa data used to estimate COPD prevalence; | |
|  |  |  | (7.80%) |  | 15,158 | 3.90% | 6,518 | 43.00% | GOLD stages II-IV | |
|  | Lesotho (7,8) | 2,192,000 | 418,672 | 43,961 | 571 | 1.30% | 410 | 71.70% | South Africa data used to estimate COPD prevalence; | |
|  |  |  | (19.10%) |  | 1,714 | 3.90% | 737 | 43.00% | BOLD study; GOLD stage II-IV | |
|  | Liberia (2,5,6) | 4,525,000 | 144,800 | 15,204 | 198 | 1.30% | 142 | 71.70% | Average of Cameroon and Algeria data used to estimate COPD prevalence; BREATHE; | |
|  |  |  | (3.20%) |  | 593 | 3.90% | 255 | 43.00% | GOLD classification (breakdown of stages not specified) | |
|  | Madagascar (6,9-11) | 24,357,000 | 1,899,846 | 199,484 | 2,593 | 1.30% | 1,859 | 71.70% | A weighted average from Eastern Africa data used to estimate COPD prevalence; | |
|  |  |  | (7.80%) |  | 7,780 | 3.90% | 3,345 | 43.00% | GOLD stages II-IV | |
|  | Malawi (6,9-11) | 17,668,000 | 1,378,104 | 144,701 | 1,881 | 1.30% | 1,349 | 71.70% | A weighted average from Eastern Africa data used to estimate COPD prevalence; | |
|  |  |  | (7.80%) |  | 5,643 | 3.90% | 2,427 | 43.00% | GOLD stages II-IV | |
|  | Mali (2,5,6) | 17,541,000 | 561,312 | 58,938 | 766 | 1.30% | 549 | 71.70% | Average of Cameroon and Algeria data used to estimate COPD prevalence; BREATHE; | |
|  |  |  | (3.20%) |  | 2,299 | 3.90% | 988 | 43.00% | GOLD classification (breakdown of stages not specified) | |
|  | Mauritania (2,5,6) | 4,205,000 | 134,560 | 14,129 | 184 | 1.30% | 132 | 71.70% | Average of Cameroon and Algeria data used to estimate COPD prevalence; BREATHE; | |
|  |  |  | (3.20%) |  | 551 | 3.90% | 237 | 43.00% | GOLD classification (breakdown of stages not specified) | |
|  | Mauritius (6,9-11) | 1,279,000 | 99,762 | 10,475 | 136 | 1.30% | 98 | 71.70% | A weighted average from Eastern Africa data used to estimate COPD prevalence; | |
|  |  |  | (7.80%) |  | 409 | 3.90% | 176 | 43.00% | GOLD stages II-IV | |
|  | Mozambique (6,9-11) | 28,165,000 | 2,196,870 | 230,671 | 2,999 | 1.30% | 2,150 | 71.70% | A weighted average from Eastern Africa data used to estimate COPD prevalence; | |
|  |  |  | (7.80%) |  | 8,996 | 3.90% | 3,868 | 43.00% | GOLD stages II-IV | |
|  | Namibia (7,8) | 2,442,000 | 466,422 | 48,974 | 637 | 1.30% | 456 | 71.70% | South Africa data used to estimate COPD prevalence; | |
|  |  |  | (19.10%) |  | 1,910 | 3.90% | 821 | 43.00% | BOLD study; GOLD stage II-IV | |
|  | Niger (2,5,6) | 19,977,000 | 639,264 | 67,123 | 873 | 1.30% | 626 | 71.70% | Average of Cameroon and Algeria data used to estimate COPD prevalence; BREATHE; | |
|  |  |  | (3.20%) |  | 2,618 | 3.90% | 1,126 | 43.00% | GOLD classification (breakdown of stages not specified) | |
|  | Nigeria (2,5,6) | 181,946,000 | 5,822,272 | 611,339 | 7,947 | 1.30% | 5,698 | 71.70% | Average of Cameroon and Algeria data used to estimate COPD prevalence; BREATHE; | |
|  |  |  | (3.20%) |  | 23,842 | 3.90% | 10,252 | 43.00% | GOLD classification (breakdown of stages not specified) | |
|  | Rwanda (6) | 11,674,000 | 1,120,704 | 117,674 | 1,530 | 1.30% | 1,097 | 71.70% | LLN criteria used, ≥45yrs | |
|  |  |  | (9.60%) |  | 4,589 | 3.90% | 1,973 | 43.00% |  |  |
|  | Senegal (2,5,6) | 15,062,000 | 481,984 | 50,608 | 658 | 1.30% | 472 | 71.70% | Average of Cameroon and Algeria data used to estimate COPD prevalence; BREATHE; | |
|  |  |  | (3.20%) |  | 1,974 | 3.90% | 849 | 43.00% | GOLD classification (breakdown of stages not specified) | |
|  | Sierra Leone (2,5,6) | 7,264,000 | 232,448 | 24,407 | 317 | 1.30% | 227 | 71.70% | Average of Cameroon and Algeria data used to estimate COPD prevalence; BREATHE; | |
|  |  |  | (3.20%) |  | 952 | 3.90% | 409 | 43.00% | GOLD classification (breakdown of stages not specified) | |
|  | Somalia (6,9-11) | 13,969,000 | 1,089,582 | 114,406 | 1,487 | 1.30% | 1,066 | 71.70% | A weighted average from Eastern Africa data used to estimate COPD prevalence; | |
|  |  |  | (7.80%) |  | 4,462 | 3.90% | 1,919 | 43.00% | GOLD stages II-IV | |
|  | South Africa (7,8) | 55,778,000 | 10,653,598 | 1,118,628 | 14,542 | 1.30% | 10,427 | 71.70% | BOLD study; | |
|  |  |  | (19.10%) |  | 43,626 | 3.90% | 18,759 | 43.00% | GOLD stage II-IV | |
|  | South Sudan (6,9-11) | 11,954,000 | 932,412 | 97,903 | 1,273 | 1.30% | 913 | 71.70% | A weighted average from Eastern Africa data used to estimate COPD prevalence; | |
|  |  |  | (7.80%) |  | 3,818 | 3.90% | 1,642 | 43.00% | GOLD stages II-IV | |
|  | Tanzania (United Republic of) (12,13) | 54,183,000 | 4,090,817 | 429,536 | 5,584 | 1.30% | 4,004 | 71.70% | GOLD stages II-IV | |
|  |  |  | (7.55%) |  | 16,752 | 3.90% | 7,203 | 43.00% |  |  |
|  | Togo (2,5,6) | 7,453,000 | 238,496 | 25,042 | 326 | 1.30% | 233 | 71.70% | Average of Cameroon and Algeria data used to estimate COPD prevalence; BREATHE; | |
|  |  |  | (3.20%) |  | 977 | 3.90% | 420 | 43.00% | GOLD classification (breakdown of stages not specified) | |
|  | Uganda (9) | 40,291,000 | 1,438,389 | 151,031 | 1,963 | 1.30% | 1,408 | 71.70% | GOLD stages II-IV | |
|  |  |  | (3.57%) |  | 5,890 | 3.90% | 2,533 | 43.00% |  |  |
|  | Zambia (6,9-11) | 16,175,000 | 1,261,650 | 132,473 | 1,722 | 1.30% | 1,235 | 71.70% | A weighted average from Eastern Africa data used to estimate COPD prevalence; | |
|  |  |  | (7.80%) |  | 5,166 | 3.90% | 2,222 | 43.00% | GOLD stages II-IV | |
|  | Zimbabwe (6,9-11) | 15,866,000 | 1,237,548 | 129,943 | 1,689 | 1.30% | 1,211 | 71.70% | A weighted average from Eastern Africa data used to estimate COPD prevalence; | |
|  |  |  | (7.80%) |  | 13,644 | 3.90% | 5,867 | 43.00% | GOLD stages II-IV | |
|  | **Totals:** | **1,197,415,000** | **64,298,051** | **6,751,295** | **87,767** | 1.30% | **62,929** | 71.70% |  | |
|  |  |  |  |  | **271,877** | 3.90% | **115,996** | 43.00% |  |  |
| **Americas** | **Latin American and the Caribbean** | | | | | | | | | |
|  | Argentina (12,14) | 44,291,000 | 3,981,761 | 418,085 | 5,435 | 1.30% | 3,897 | 71.70% | GOLD stages II-IV | |
|  |  |  | (8.99%) |  | 16,305 | 3.90% | 7,011 | 43.00% |  |  |
|  | Bolivia (Plurinational State of) (8) | 10,852,000 | 765,066 | 80,332 | 1,044 | 1.30% | 749 | 71.70% | Chile data used to estimate COPD prevalence; | |
|  |  |  | (7.05%) |  | 3,133 | 3.90% | 1,347 | 43.00% | BOLD study; GOLD stages II-IV | |
|  | Brazil (8,13) | 209,009,000 | 12,645,045 | 1,327,730 | 17,260 | 1.30% | 12,376 | 71.70% | BOLD study; | |
|  |  |  | (6.05%) |  | 51,781 | 3.90% | 22,266 | 43.00% | GOLD stages II-IV | |
|  | Chile (8) | 18,089,000 | 1,275,275 | 133,904 | 1,741 | 1.30% | 1,248 | 71.70% | BOLD study; | |
|  |  |  | (7.05%) |  | 5,222 | 3.90% | 2,246 | 43.00% | GOLD stages II-IV | |
|  | Colombia (15,16) | 48,801,000 | 1,351,788 | 141,938 | 1,845 | 1.30% | 1,323 | 71.70% | GOLD stages II-IV | |
|  |  |  | (2.77%) |  | 5,536 | 3.90% | 2,380 | 43.00% |  |  |
|  | Costa Rica (8) | 4,882,000 | 151,342 | 15,891 | 207 | 1.30% | 148 | 71.70% | Mexico data used to estimate COPD prevalence; | |
|  |  |  | (3.10%) |  | 620 | 3.90% | 266 | 43.00% | BOLD study; GOLD stages II-IV | |
|  | Cuba (17) | 11,773,000 | 1,659,993 | 174,299 | 2,266 | 1.30% | 1,625 | 71.70% | Jamaica data used to estimate COPD prevalence; COPD classification system not specified | |
|  |  |  | (14.10%) |  | 6,798 | 3.90% | 2,923 | 43.00% |  |  |
|  | Dominican Republic (18) | 10,658,000 | 1,502,778 | 157,792 | 2,051 | 1.30% | 1,471 | 71.70% | Jamaica data used to estimate COPD prevalence; COPD classification system not specified | |
|  |  |  | (14.10%) |  | 6,154 | 3.90% | 2,646 | 43.00% |  |  |
|  | Ecuador (8,18) | 16,344,000 | 1,152,252 | 120,986 | 1,573 | 1.30% | 1,128 | 71.70% | Chile data used to estimate COPD prevalence; | |
|  |  |  | (7.05%) |  | 4,718 | 3.90% | 2,029 | 43.00% | BOLD study; GOLD stages II-IV | |
|  | El Salvador (8) | 6,414,000 | 198,834 | 20,878 | 271 | 1.30% | 195 | 71.70% | Mexico data used to estimate COPD prevalence; | |
|  |  |  | (3.10%) |  | 814 | 3.90% | 350 | 43.00% | BOLD study; GOLD stages II-IV | |
|  | Guatemala (8) | 16,395,000 | 508,245  (3.10%) | 53,366 | 694 | 1.30% | 497 | 71.70% | Mexico data used to estimate COPD prevalence;  BOLD study; GOLD stages II-IV | |
|  |  |  |  |  | 2,081 | 3.90% | 895 | 43.00% |  |  |
|  | Haiti (17) | 10,807,000 | 1,523,787 | 159,998 | 2,080 | 1.30% | 1,491 | 71.70% | Jamaica data used to estimate COPD prevalence; COPD classification system not specified | |
|  |  |  | (14.10%) |  | 6,240 | 3.90% | 2,683 | 43.00% |  |  |
|  | Honduras (8) | 9,032,000 | 279,992 | 29,399 | 382 | 1.30% | 274 | 71.70% | Mexico data used to estimate COPD prevalence; | |
|  |  |  | (3.10%) |  | 1,147 | 3.90% | 493 | 43.00% | BOLD study; GOLD stages II-IV | |
|  | Jamaica (17) | 2,923,000 | 412,143 | 43,275 | 563 | 1.30% | 403 | 71.70% | COPD classification system not specified | |
|  |  |  | (14.10%) |  | 1,688 | 3.90% | 726 | 43.00% |  |  |
|  | Mexico (8) | 127,322,000 | 3,946,982 | 414,433 | 5,388 | 1.30% | 3,863 | 71.70% | BOLD study; | |
|  |  |  | (3.10%) |  | 16,163 | 3.90% | 6,950 | 43.00% | GOLD stages II-IV | |
|  | Nicaragua (8) | 6,140,000 | 190,340 | 19,986 | 260 | 1.30% | 186 | 71.70% | Mexico data used to estimate COPD prevalence; | |
|  |  |  | (3.10%) |  | 779 | 3.90% | 335 | 43.00% | BOLD study; GOLD stages II-IV | |
|  | Panama (8) | 4,023,000 | 52,299 | 5,491 | 71 | 1.30% | 51 | 71.70% | Mexico data used to estimate COPD prevalence; | |
|  |  |  | (3.10%) |  | 214 | 3.90% | 92 | 43.00% | BOLD study; GOLD stages II-IV | |
|  | Paraguay (8) | 6,713,000 | 406,137 | 42,644 | 554 | 1.30% | 397 | 71.70% | Brazil data used to estimate COPD prevalence; | |
|  |  |  | (6.05%) |  | 1,663 | 3.90% | 715 | 43.00% | BOLD study; GOLD stages II-IV | |
|  | Peru (19) | 31,776,000 | 565,613 | 59,389 | 772 | 1.30% | 554 | 71.70% | GOLD stages II-IV | |
|  |  |  | (1.78%) |  | 2,316 | 3.90% | 996 | 43.00% |  |  |
|  | Puerto Rico (17) | 3,769,000 | 531,429 | 55,800 | 725 | 1.30% | 520 | 71.70% | Jamaica data used to estimate COPD prevalence; COPD classification system not specified | |
|  |  |  | (14.10%) |  | 2,176 | 3.90% | 936 | 43.00% |  |  |
|  | Trinidad and Tobago (17) | 1,381,000 | 194,721 | 20,446 | 266 | 1.30% | 191 | 71.70% | Jamaica data used to estimate COPD prevalence; COPD classification system not specified | |
|  |  |  | (14.10%) |  | 797 | 3.90% | 343 | 43.00% |  |  |
|  | Uruguay (20) | 3,526,000 | 250,346 | 26,286 | 342 | 1.30% | 245 | 71.70% | PLATINO study; | |
|  |  |  | (7.10%) |  | 1,025 | 3.90% | 441 | 43.00% | GOLD stages II-IV | |
|  | Venezuela (Bolivarian Republic of) (20) | 31,497,000 | 1,795,329 | 188,510 | 2,451 | 1.30% | 1,757 | 71.70% | PLATINO study; | |
|  |  |  | (5.70%) |  | 7,352 | 3.90% | 3,161 | 43.00% | GOLD stages II-IV | |
|  | **Northern America** | | | | | | | | | |
|  | Canada (7) | 36,939,000 | 3,028,998 | 318,045 | 4,135 | 1.30% | 2,964 | 71.70% | BOLD study; | |
|  |  |  | (8.20%) |  | 12,404 | 3.90% | 5,334 | 43.00% | GOLD stages II-IV | |
|  | United States of America (8) | 328,030,000 | 46,908,290 | 4,925,370 | 64,030 | 1.30% | 45,909 | 71.70% | BOLD study; | |
|  |  |  | (14.30%) |  | 192,089 | 3.90% | 82,598 | 43.00% | GOLD stages II-IV | |
|  | **Totals:** | **1,001,386,000** | **85,278,783** | **8,954,272** | **11,6406** | 1.30% | **83,463** | 71.70% |  | |
|  |  |  |  |  | **349,217** | 3.90% | **15,0163** | 43.00% |  |  |
| **Asia** | **Central Asia** | | | | | | | | | |
|  | Kazakhstan (21) | 18,074,000 | 777,182 | 81,604 | 1,061 | 1.30% | 761 | 71.70% | GOLD (stages not specified) | |
|  |  |  | (4.30%) |  | 3,183 | 3.90% | 1,369 | 43.00% | Data for EURO WHO region | |
|  | Kyrgyzstan (22) | 5,924,000 | 485,768 | 51,006 | 663 | 1.30% | 475 | 71.70% | GOLD stages II-IV | |
|  |  |  | (8.20%) |  | 1,989 | 3.90% | 855 | 43.00% |  |  |
|  | Tajikistan (23) | 8,612,000 | 1,179,844 | 123,884 | 1,610 | 1.30% | 1,155 | 71.70% | GOLD (stages not specified) | |
|  |  |  | (13.70%) |  | 4,831 | 3.90% | 2,078 | 43.00% | Data for EURO WHO region | |
|  | Turkmenistan (23) | 5,613,000 | 768,981 | 80,743 | 1,050 | 1.30% | 753 | 71.70% | GOLD (stages not specified) | |
|  |  |  | (13.70%) |  | 3,149 | 3.90% | 1,354 | 43.00% | Data for EURO WHO region | |
|  | Uzbekistan (23) | 31,261,000 | 4,282,757 | 449,689 | 5,846 | 1.30% | 4,192 | 71.70% | GOLD (stages not specified) | |
|  |  |  | (13.70%) |  | 17,538 | 3.90% | 7,541 | 43.00% | Data for EURO WHO region | |
|  | **Eastern Asia** | | | | | | | | | |
|  | China (7,24-26) | 1,421,905,000 | 82,754,871 | 8,689,261 | 112,960 | 1.30% | 80,993 | 71.70% | BOLD study; | |
|  |  |  | (5.82%) |  | 338,881 | 3.90% | 145,719 | 43.00% | GOLD stages II-IV | |
|  | Hong Kong (7,24-26) | 7,460,000 | 434,172 | 45,588 | 470 | 1.30% | 337 | 71.70% | China data used to estimate COPD prevalence; | |
|  |  |  | (5.82%) |  | 1,778 | 3.90% | 765 | 43.00% | BOLD study; GOLD stages II-IV | |
|  | Japan (27) | 134,230,000 | 6,443,040 | 676,519 | 8,795 | 1.30% | 6,306 | 71.70% | GOLD stages II-IV | |
|  |  |  | (4.80%) |  | 26,384 | 3.90% | 11,345 | 43.00% |  |  |
|  | Korea (Democratic People's Republic of) (28) | 25,736,000 | 3,448,624 | 362,106 | 4,707 | 1.30% | 3,375 | 71.70% | South Korea data used to estimate COPD prevalence; | |
|  |  |  | (13.40%) |  | 14,122 | 3.90% | 6,073 | 43.00% | FEV1 <0.7 | |
|  | Korea (Republic of) (28) | 51,926,000 | 6,958,084 | 730,599 | 9,498 | 1.30% | 6,810 | 71.70% | FEV1 <0.7 | |
|  |  |  | (13.40%) |  | 28,493 | 3.90% | 12,252 | 43.00% |  |  |
|  | Mongolia (7,24-26) | 2,999,000 | 174,542 | 18,327 | 238 | 1.30% | 171 | 71.70% | China data used to estimate COPD prevalence; | |
|  |  |  | (5.82%) |  | 715 | 3.90% | 307 | 43.00% | BOLD study; GOLD stages II-IV | |
|  | Taiwan, province of China (7,24-26) | 24,042,000 | 1,399,244 | 146,921 | 1,910 | 1.30% | 1,369 | 71.70% | China data used to estimate COPD prevalence; | |
|  |  |  | (5.82%) |  | 5,730 | 3.90% | 2,464 | 43.00% | BOLD study; GOLD stages II-IV | |
|  | **South-Eastern Asia** | | | | | | | | | |
|  | Cambodia (29) | 15,628,000 | 578,236 | 6,0715 | 789 | 1.30% | 566 | 71.70% | Vietnam data used to estimate COPD prevalence; | |
|  |  |  | (3.70) |  | 2,368 | 3.90% | 1,018 | 43.00% | GOLD stages II-IV | |
|  | Indonesia (8) | 260,597,000 | 32,574,625 | 3,420,336 | 44,464 | 1.30% | 31,881 | 71.70% | Philippines data used to estimate COPD prevalence; | |
|  |  |  | (12.50%) |  | 133,393 | 3.90% | 57,359 | 43.00% | BOLD study; GOLD stages II-IV | |
|  | Lao People's Democratic Republic (29) | 6,712,000 | 248,344 | 26,076 | 339 | 1.30% | 243 | 71.70% | Vietnam data used to estimate COPD prevalence; | |
|  |  |  | (3.70%) |  | 1,017 | 3.90% | 437 | 43.00% | GOLD stages II-IV | |
|  | Malaysia (30) | 31,049,000 | 1,288,534 | 135,296 | 1,759 | 1.30% | 1,261 | 71.70% | Thailand data used to estimate COPD prevalence; | |
|  |  |  | (4.15%) |  | 5,277 | 3.90% | 2,269 | 43.00% | GOLD stages II-IV | |
|  | Myanmar (30) | 52,885,000 | 2,194,728 | 230,446 | 2,996 | 1.30% | 2,148 | 71.70% | Thailand data used to estimate COPD prevalence; | |
|  |  |  | (4.15%) |  | 8,987 | 3.90% | 3,865 | 43.00% | GOLD stages II-IV | |
|  | Philippines (8) | 102,565,000 | 12,820,625 | 1,346,166 | 17,500 | 1.30% | 12,548 | 71.70% | BOLD study; | |
|  |  |  | (12.50%) |  | 52,500 | 3.90% | 22,575 | 43.00% | GOLD stages II-IV | |
|  | Singapore (29) | 5,651,000 | 209,087 | 21,954 | 285 | 1.30% | 205 | 71.70% | Vietnam data used to estimate COPD prevalence; | |
|  |  |  | (3.70%) |  | 856 | 3.90% | 368 | 43.00% | GOLD stages II-IV | |
|  | Thailand (30) | 70,050,000 | 2,907,075 | 305,243 | 3,968 | 1.30% | 2,845 | 71.70% | GOLD stages II-IV | |
|  |  |  | (4.15%) |  | 11,904 | 3.90% | 5,119 | 43.00% |  |  |
|  | Timor-Leste (8) | 1,247,000 | 155,875 | 16,367 | 213 | 1.30% | 153 | 71.70% | Philippines data used to estimate COPD prevalence; | |
|  |  |  | (12.50%) |  | 638 | 3.90% | 274 | 43.00% | BOLD study; GOLD stages II-IV | |
|  | Vietnam (29) | 94,734,000 | 3,505,158 | 368,042 | 4,785 | 1.30% | 3,431 | 71.70% | GOLD stages II-IV | |
|  |  |  | (3.70%) |  | 14,354 | 3.90% | 6,172 | 43.00% |  |  |
|  | **Southern Asia** | | | | | | | | | |
|  | Afghanistan (31) | 33,866,000 | 4,673,508 | 490,718 | 6,379 | 1.30% | 4,574 | 71.70% | Pakistan data used to estimate COPD prevalence; | |
|  |  |  | (13.80%) |  | 19,138 | 3.90% | 8,229 | 43.00% | GOLD stages not specified | |
|  | Bangladesh (32) | 162,678,000 | 17,569,224 | 1,844,769 | 23,982 | 1.30% | 17,195 | 71.70% | GOLD stages II-IV | |
|  |  |  | (10.80%) |  | 71,946 | 3.90% | 30,937 | 43.00% |  |  |
|  | India (33) | 1,322,312,000 | 93,884,152 | 9,857,836 | 128,152 | 1.30% | 91,885 | 71.70% | GOLD stage breakdown not specified | |
|  |  |  | (7.10%) |  | 384,456 | 3.90% | 165,316 | 43.00% |  |  |
|  | Iran (Islamic Republic of) (34) | 80,142,000 | 7,373,064 | 774,172 | 10,064 | 1.30% | 7,216 | 71.70% | GOLD stage breakdown not specified | |
|  |  |  | (9.20%) |  | 30,193 | 3.90% | 12,983 | 43.00% |  |  |
|  | Nepal (7,24-26) | 28,930,000 | 1,683,726 | 176,791 | 2,298 | 1.30% | 1,648 | 71.70% | China data used to estimate COPD prevalence; | |
|  |  |  | (5.82%) |  | 6,895 | 3.90% | 2,965 | 43.00% | BOLD study; GOLD stages II-IV | |
|  | Pakistan (31) | 190,944,000 | 26350272 | 2766779 | 35,968 | 1.30% | 25,789 | 71.70% | GOLD stages not specified | |
|  |  |  | (13.80%) |  | 107,904 | 3.90% | 46,399 | 43.00% |  | |
|  | Sri Lanka (33) | 20,995,000 | 1490645 | 156518 | 2,035 | 1.30% | 1,459 | 71.70% | India data used to estimate COPD prevalence; | |
|  |  |  | (7.10%) |  | 6,104 | 3.90% | 2,625 | 43.00% | GOLD stage breakdown not specified | |
|  | **Western Asia** | | | | | | | | | |
|  | Armenia (23) | 2,926,000 | 397,936 | 41,783 | 543 | 1.30% | 389 | 543 | GOLD (stages not specified)  Data for EURO WHO region | |
|  |  |  | (13.6%) |  | 1,630 | 3.90% | 701 | 1,630 |  |  |
|  | Azerbaijan (23) | 9,756,000 | 1,336,572 | 140,340 | 1,824 | 1.30% | 1,308 | 71.70% | GOLD (stages not specified) | |
|  |  |  | (13.70%) |  | 5,473 | 3.90% | 2,354 | 43.00% | Data for EURO WHO region | |
|  | Bahrain (2,5,35) | 1,376,000 | 41,968 | 4,407 | 57 | 1.30% | 41 | 71.70% | Saudi Arabia data used to estimate COPD prevalence; | |
|  |  |  | (3.05%) |  | 172 | 3.90% | 74 | 43.00% | BREATHE (epidemiological survey) and GOLD (stages II-IV) | |
|  | Cyprus (36) | 1,192,000 | 36,952 | 3880 | 50 | 1.30% | 36 | 71.70% | GOLD stages II-IV | |
|  |  |  | (3.10%) |  | 151 | 3.90% | 65 | 43.00% |  |  |
|  | Georgia (23) | 4,099,000 | 561,563 | 58964 | 767 | 1.30% | 550 | 71.70% | GOLD (stages not specified) | |
|  |  |  | (13.70%) |  | 2,300 | 3.90% | 989 | 43.00% | Data for EURO WHO region | |
|  | Iraq (37) | 36,327,000 | 4,915,043 | 516080 | 6,709 | 1.30% | 4,810 | 71.70% | GOLD stage II and III | |
|  |  |  | (13.53%) |  | 20,127 | 3.90% | 8,655 | 43.00% |  |  |
|  | Israel (2,5) | 8,222,000 | 443,988 | 46,619 | 606 | 1.30% | 435 | 71.70% | Jordan data used to estimate COPD prevalence; | |
|  |  |  | (5.40%) |  | 1,818 | 3.90% | 782 | 43.00% | BREATHE study; Epidemiological survey | |
|  | Jordan (2,5) | 9,224,000 | 498,096 | 52300 | 680 | 1.30% | 487 | 71.70% | BREATHE study; | |
|  |  |  | (5.40%) |  | 2,040 | 3.90% | 877 | 43.00% | Epidemiological survey | |
|  | Kuwait (37) | 3,950,000 | 534,435 | 56,116 | 730 | 1.30% | 523 | 71.70% | Iraq data used to estimate COPD prevalence; | |
|  |  |  | (13.53%) |  | 2,189 | 3.90% | 941 | 43.00% | GOLD stage II and III | |
|  | Lebanon (2,5) | 5,952,000 | 315,456 | 33,123 | 431 | 1.30% | 309 | 71.70% | BREATHE study; | |
|  |  |  | (5.30%) |  | 1,292 | 3.90% | 555 | 43.00% | Epidemiological survey | |
|  | Oman (2,5,35) | 4,219,000 | 128,680 | 13,511 | 176 | 1.30% | 126 | 71.70% | Saudi Arabia data used to estimate COPD prevalence; | |
|  |  |  | (3.05%) |  | 527 | 3.90% | 227 | 43.00% | BREATHE study (epidemiological survey) and GOLD (stages II-IV) | |
|  | Palestine (State of) (2,5) | 4,688,000 | 164,080 | 17,228 | 224 | 1.30% | 161 | 71.70% | Egypt data used to estimate COPD prevalence; BREATHE study; | |
|  |  |  | (3.50%) |  | 672 | 3.90% | 289 | 43.00% | Epidemiological survey | |
|  | Qatar (2,5,35) | 2,488,000 | 75,884 | 7,968 | 104 | 1.30% | 74 | 71.70% | Saudi Arabia data used to estimate COPD prevalence; | |
|  |  |  | (3.05%) |  | 311 | 3.90% | 134 | 43.00% | BREATHE study (epidemiological survey) and GOLD (stages II-IV) | |
|  | Saudi Arabia (2,5,35) | 31,722,000 | 967,521 | 101,590 | 1,321 | 1.30% | 947 | 71.70% | BREATHE study (epidemiological survey) and GOLD (stages II-IV) | |
|  |  |  | (3.05%) |  | 3,962 | 3.90% | 1,704 | 43.00% |  |  |
|  | Syrian Arab Republic (2,5) | 18,877,000 | 1,151,497 | 120,907 | 1,572 | 1.30% | 1,127 | 71.70% | BREATHE study; | |
|  |  |  | (6.10%) |  | 4,715 | 3.90% | 2,028 | 43.00% | Epidemiological survey | |
|  | Turkey (8) | 79,456,000 | 8,422,336 | 884345 | 11,496 | 1.30% | 8,243 | 71.70% | BOLD study; GOLD stages II-IV; | |
|  |  |  | (10.60%) |  | 34,489 | 3.90% | 14,830 | 43.00% | BREATHE study; Epidemiological survey | |
|  | United Arab Emirates (2,5) | 9,164,000 | 174,116 | 18,282 | 238 | 1.30% | 170 | 71.70% | BREATHE study; | |
|  |  |  | (1.90%) |  | 713 | 3.90% | 307 | 43.00% | Epidemiological survey | |
|  | Yemen (2,5,35) | 27,040,000 | 824,720 | 86,596 | 1,126 | 1.30% | 807 | 71.70% | Saudi Arabia data used to estimate COPD prevalence; | |
|  |  |  | (3.05%) |  | 3,377 | 3.90% | 1,452 | 43.00% | BREATHE study (epidemiological survey) and GOLD (stages II-IV) | |
|  | **Totals:** | **4,476,519,000** | **339,206,893** | **35,616,724** | **462,894** | 1.30% | **331,895** | 71.70% |  | |
|  |  |  |  |  | **1,389,052** | 3.90% | **597,292** | 43.00% |  |  |
| **Europe** | **Eastern Europe** | | | | | | | | |  |
|  | Belarus (7) | 9,804,000 | 1,068,636 | 112,207 | 1,459 | 1.30% | 1,046 | 71.70% | Poland data used to estimate COPD prevalence; |  |
|  |  |  | (10.90%) |  | 4,376 | 3.90% | 1,882 | 43.00% | BOLD study; GOLD stages II-IV |  |
|  | Bulgaria (23) | 7,450,000 | 1,020,650 | 107,168 | 1,393 | 1.30% | 999 | 71.70% | GOLD (stages not specified) |  |
|  |  |  | (13.70%) |  | 4,180 | 3.90% | 1,797 | 43.00% | Data for EURO WHO region |  |
|  | Czech Republic (7) | 10,915,000 | 1,189,735 | 124,922 | 1,624 | 1.30% | 1,164 | 71.70% | Poland data used to estimate COPD prevalence; |  |
|  |  |  | (10.90%) |  | 4,872 | 3.90% | 2,095 | 43.00% | BOLD study; GOLD stages II-IV |  |
|  | Hungary (7) | 10,113,000 | 1,071,978 | 112,558 | 1,463 | 1.30% | 1,049 | 71.70% | Austria data used to estimate COPD prevalence; |  |
|  |  |  | (10.60%) |  | 4,390 | 3.90% | 1,888 | 43.00% | BOLD study; GOLD stages II-IV |  |
|  | Moldova (Republic of) (23) | 4,153,000 | 568,961 | 59,741 | 777 | 1.30% | 557 | 71.70% | GOLD (stages not specified) |  |
|  |  |  | (13.70%) |  | 2,330 | 3.90% | 1,002 | 43.00% | Data for EURO WHO region |  |
|  | Poland (8) | 39,416,000 | 4,296,344 | 451,116 | 5,865 | 1.30% | 4,205 | 71.70% | BOLD study; |  |
|  |  |  | (10.90%) |  | 17,594 | 3.90% | 7,565 | 43.00% | GOLD stages II-IV |  |
|  | Romania (38) | 20,635,000 | 1,919,055 | 201,501 | 2,620 | 1.30% | 1,878 | 71.70% | GOLD (stages not specified) |  |
|  |  |  | (9.30%) |  | 7,859 | 3.90% | 3,379 | 43.00% |  |  |
|  | Russian Federation (39) | 148,880,000 | 7146240 | 750355 | 9,755 | 1.30% | 6,994 | 71.70% | LLN |  |
|  |  |  | (4.80%) |  | 29,264 | 3.90% | 12,583 | 43.00% |  |  |
|  | Slovakia (8) | 5,575,000 | 607,675 | 63,806 | 829 | 1.30% | 595 | 71.70% | Poland data used to estimate COPD prevalence; |  |
|  |  |  | (10.90%) |  | 2,488 | 3.90% | 1,070 | 43.00% | BOLD study; GOLD stages II-IV |  |
|  | Ukraine (23) | 46,502,000 | 6,370,774 | 668,931 | 8,696 | 1.30% | 6,235 | 71.70% | GOLD (stages not specified) |  |
|  |  |  | (13.70%) |  | 26,088 | 3.90% | 11,218 | 43.00% | Data for EURO WHO region |  |
|  | **Northern Europe** | | | | | | | | |  |
|  | Denmark (40,41) | 5,878,000 | 734,750 | 77149 | 1,003 | 1.30% | 5,698 | 71.70% | GOLD stages II-IV |  |
|  |  |  | (12.65%) |  | 3,009 | 3.90% | 1,294 | 43.00% |  |  |
|  | Estonia (39) | 1,369,000 | 65,712 | 6,900 | 90 | 1.30% | 64 | 71.70% | Russia data used to estimate COPD prevalence; |  |
|  |  |  | (4.80%) |  | 269 | 3.90% | 116 | 43.00% | LLN |  |
|  | Finland (42-44) | 5,677,000 | 313938 | 32,964 | 429 | 1.30% | 307 | 71.70% | GOLD stages II-IV |  |
|  |  |  | (5.53%) |  | 1,286 | 3.90% | 553 | 43.00% |  |  |
|  | Ireland (40,45) | 4,812,000 | 457,140 | 48,000 | 624 | 1.30% | 447 | 71.70% | UK data used to estimate COPD prevalence; |  |
|  |  |  | (9.50%) |  | 1,872 | 3.90% | 805 | 43.00% | GOLD stages II-IV |  |
|  | Latvia (39) | 2,080,000 | 99,840 | 10,483 | 136 | 1.30% | 98 | 71.70% | Russia data used to estimate COPD prevalence; |  |
|  |  |  | (4.80%) |  | 409 | 3.90% | 176 | 43.00% | LLN |  |
|  | Lithuania (39) | 3,054,000 | 146,592 | 15,392 | 200 | 1.30% | 143 | 71.70% | Russia data used to estimate COPD prevalence; |  |
|  |  |  | (4.80%) |  | 4,756 | 3.90% | 2,045 | 43.00% | LLN |  |
|  | Norway (8) | 5,340,000 | 443,220 | 46,538 | 605 | 1.30% | 434 | 71.70% | BOLD study; |  |
|  |  |  | (8.30%) |  | 1,815 | 3.90% | 780 | 43.00% | GOLD stages II-IV |  |
|  | Sweden (40,46) | 10,093,000 | 817533 | 85,841 | 1,116 | 1.30% | 800 | 71.70% | GOLD stages II-IV |  |
|  |  |  | (8.10%) |  | 3,348 | 3.90% | 1,440 | 43.00% |  |  |
|  | United Kingdom and Northern Ireland (40,45,47) | 67,582,000 | 6,420,290 | 674,130 | 8,764 | 1.30% | 6,284 | 71.70% | GOLD stages II-IV |  |
|  |  |  | (9.50%) |  | 26,291 | 3.90% | 11,305 | 43.00% |  |  |
|  | **Southern Europe** | | | | | | | | |  |
|  | Albania (48,49) | 3,007,000 | 264,616 | 27,785 | 361 | 1.30% | 259 | 71.70% | Greece data used to estimate COPD prevalence; |  |
|  |  |  | (8.80%) |  | 1,084 | 3.90% | 466 | 43.00% | GOLD stages II-IV |  |
|  | Bosnia and Herzegovina (48,49) | 3,671,000 | 323,048 | 33,920 | 441 | 1.30% | 316 | 71.70% | Greece data used to estimate COPD prevalence; |  |
|  |  |  | (8.80%) |  | 1,323 | 3.90% | 569 | 43.00% | GOLD stages II-IV |  |
|  | Croatia (50,51) | 4,415,000 | 291,390 | 30,596 | 398 | 1.30% | 285 | 71.70% | Italy data used to estimate COPD prevalence; |  |
|  |  |  | (6.60%) |  | 1,193 | 3.90% | 513 | 43.00% | GOLD stages II-IV |  |
|  | Greece (48,49) | 11,699,000 | 1,029,512 | 108,099 | 1,405 | 1.30% | 1,008 | 71.70% | GOLD stages II-IV |  |
|  |  |  | (8.80%) |  | 4,216 | 3.90% | 1,813 | 43.00% |  |  |
|  | Italy (50,51) | 62,261,000 | 4,109,226 | 431,469 | 5,609 | 1.30% | 4,022 | 71.70% | GOLD stages II-IV |  |
|  |  |  | (6.60%) |  | 16,827 | 3.90% | 7,236 | 43.00% |  |  |
|  | Macedonia (the former Yugoslav Republic of) (48,49) | 2,133,000 | 187,704 | 19,709 | 256 | 1.30% | 184 | 71.70% | Greece data used to estimate COPD prevalence; | |
|  |  |  | (8.80%) |  | 769 | 3.90% | 331 | 43.00% | GOLD stages II-IV | |
|  | Portugal (52) | 10,857,000 | 792,561 | 83,219 | 1,082 | 1.30% | 776 | 71.70% | GOLD stages II-IV | |
|  |  |  | (7.30%) |  | 3,246 | 3.90% | 1,396 | 43.00% |  |  |
|  | Serbia (53) | 9,136,000 | 324,328 | 34,054 | 443 | 1.30% | 317 | 71.70% | GOLD (stages not specified) | |
|  |  |  | (3.55%) |  | 1,328 | 3.90% | 571 | 43.00% |  |  |
|  | Slovenia (7) | 2,149,000 | 227,794 | 23,918 | 311 | 1.30% | 223 | 71.70% | Austria data used as estimate for COPD prevalence; | |
|  |  |  | (10.60%) |  | 933 | 3.90% | 401 | 43.00% | BOLD study; GOLD stages II-IV | |
|  | Spain (54,55) | 48,009,000 | 2,520,473 | 264,650 | 3,440 | 1.30% | 2,467 | 71.70% | GOLD stages II-IV | |
|  |  |  | (5.25%) |  | 10,321 | 3.90% | 4,438 | 43.00% |  |  |
|  | **Western Europe** | | | | | | | | | |
|  | Austria (7) | 8,999,000 | 953,894 | 100,159 | 1,302 | 1.30% | 934 | 71.70% | BOLD study; | |
|  |  |  | (10.60%) |  | 3,906 | 3.90% | 1,680 | 43.00% | GOLD stages II-IV | |
|  | Belgium (56) | 11,686,000 | 1,215,344 | 127,611 | 1,659 | 1.30% | 1,189 | 71.70% | Netherlands data used to estimate COPD prevalence; GOLD stages II-IV | |
|  |  |  | (10.40%) |  | 4,977 | 3.90% | 2,140 | 43.00% |  |  |
|  | France (57) | 66,627,000 | 4,997,025 | 524,688 | 6,821 | 1.30% | 4,891 | 71.70% | GOLD stages II-IV | |
|  |  |  | (7.50%) |  | 20,463 | 3.90% | 8,799 | 43.00% |  |  |
|  | Germany (7) | 85,901,000 | 5,068,159 | 532,157 | 6,918 | 1.30% | 4,960 | 71.70% | BOLD study; | |
|  |  |  | (5.90%) |  | 20,754 | 3.90% | 8,924 | 43.00% | GOLD stages II-IV | |
|  | Netherlands (56) | 17,488,000 | 1,818,752 | 190,969 | 2,483 | 1.30% | 1,780 | 71.70% | GOLD stages II-IV | |
|  |  |  | (10.40%) |  | 7,448 | 3.90% | 3,203 | 43.00% |  |  |
|  | Switzerland (58) | 8,592,000 | 601,440 | 63,151 | 821 | 1.30% | 589 | 71.70% | GOLD stages II-IV | |
|  |  |  | (7.00%) |  | 2,463 | 3.90% | 1,059 | 43.00% |  |  |
|  | **Totals:** | **765,958,000** | **59,484,329** | **6,245,855** | **88,139** | 1.30% | **63,196** | 71.70% |  | |
|  |  |  |  |  | **247,744** | 3.90% | **106,530** | 43.00% |  |  |
| **Oceania** | **Oceania** | | | | | | | | | |
|  | Australia (7) | 24,429,000 | 2,638,332 | 277,025 | 3,601 | 1.30% | 2,582 | 71.70% | BOLD study; | |
|  |  |  | (10.80%) |  | 10,804 | 3.90% | 4,646 | 43.00% | GOLD stages II-IV | |
|  | New Zealand (59) | 4,732,000 | 397,961 | 41,786 | 543 | 1.30% | 389 | 71.70% | GOLD stages II-IV | |
|  |  |  | (8.41%) |  | 1,630 | 3.90% | 701 | 43.00% |  |  |
|  | Papua New Guinea (8) | 7,970,000 | 996,250 | 104,606 | 1,360 | 1.30% | 975 | 71.70% | Philippines data used to estimate COPD prevalence; | |
|  |  |  | (12.50%) |  | 4,080 | 3.90% | 1,754 | 43.00% | BOLD study; GOLD stages II-IV | |
|  | **Totals:** | **37,131,000** | **4,032,543** | **423,417** | **5,504** | 1.30% | **3,947** | 71.70% |  | |
|  |  |  |  |  | **16,513** | 3.90% | **7,101** | 43.00% |  |  |

*LLN = lower limit of normal*

**References for Table S1**

| 1. UN 2015 population data |
| --- |
| 1. Polatli, M., Ben Kheder, A., Wali, S., Javed, A., Khattab, A., Mahboub, B., Iraqi, G., Nejjari, C., Taright, S., Koniski, M., Rashid, N. and El Hasnaoui, A. (2012). Chronic obstructive pulmonary disease and associated healthcare resource consumption in the Middle East and North Africa: The BREATHE study. *Respiratory Medicine*, 106, pp.S75-S85. |
| 1. Guinea, J., Torres-Narbona, M., Gijón, P., Muñoz, P., Pozo, F., Peláez, T., de Miguel, J. and Bouza, E. (2010). Pulmonary aspergillosis in patients with chronic obstructive pulmonary disease: incidence, risk factors, and outcome. *Clinical Microbiology and Infection*, 16(7), pp.870-877. |
| 1. Xu, H., Li, L., Huang, W., Wang, L., Li, W. and Yuan, W. (2012). Invasive pulmonary aspergillosis in patients with chronic obstructive pulmonary disease: a case control study from China. *Clinical Microbiology and Infection*, 18(4), pp.403-408. |
| 1. Tageldin, M., Nafti, S., Khan, J., Nejjari, C., Beji, M., Mahboub, B., Obeidat, N., Uzaslan, E., Sayiner, A., Wali, S., Rashid, N. and El Hasnaoui, A. (2012). Distribution of COPD-related symptoms in the Middle East and North Africa: Results of the BREATHE study. Respiratory Medicine, 106, pp.S25-S32. |
| 1. Musafiri, S., van Meerbeeck, J., Musango, L., Brusselle, G., Joos, G., Seminega, B. and Rutayisire, C. (2011). Prevalence of atopy, asthma and COPD in an urban and a rural area of an African country. Respiratory Medicine, 105(11), pp.1596-1605. |
| 1. Buist AS, McBurnie MA, Vollmer WM, et al. International variation in the prevalence of COPD (The BOLD Study): a population-based prevalence study. *Lancet*. 2007;370(9589):741-750. doi:10.1016/S0140-6736(07)61377-4 |
| 1. Mannino DM, Buist AS. Global burden of COPD: risk factors, prevalence, and future trends. *Lancet*. 2007;370(9589):765-773. doi:10.1016/S0140-6736(07)61380-4 |
| 1. van Gemert, F., Kirenga, B., Chavannes, N., Kamya, M., Luzige, S., Musinguzi, P., Turyagaruka, J., Jones, R., Tsiligianni, I., Williams, S., de Jong, C. and van der Molen, T. (2015). Prevalence of chronic obstructive pulmonary disease and associated risk factors in Uganda (FRESH AIR Uganda): a prospective cross-sectional observational study. The Lancet Global Health, 3(1), pp.e44-e51. |
| 1. Magitta, N., Walker, R., Apte, K., Shimwela, M., Mwaiselage, J., Sanga, A., Namdeo, A., Madas, S. and Salvi, S. (2018). Prevalence, risk factors and clinical correlates of COPD in a rural setting in Tanzania. European Respiratory Journal, 51(2), p.1700182. |
| 1. Daldoul, H., Denguezli, M., Jithoo, A., Gnatiuc, L., Buist, S., Burney, P., Tabka, Z. and Harrabi, I. (2013). Prevalence of COPD and Tobacco Smoking in Tunisia — Results from the BOLD Study. International Journal of Environmental Research and Public Health, 10(12), pp.7257-7271. |
| 1. Echazarreta, A., Arias, S., del Olmo, R., Giugno, E., Colodenco, F., Arce, S., Bossio, J., Armando, G. and Soriano, J. (2018). Prevalence of COPD in 6 Urban Clusters in Argentina: The EPOC.AR Study. Archivos de Bronconeumología (English Edition), 54(5), pp.260-269. |
| 1. Foo, J., Landis, S., Maskell, J., Oh, Y., van der Molen, T., Han, M., Mannino, D., Ichinose, M. and Punekar, Y. (2016). Continuing to Confront COPD International Patient Survey: Economic Impact of COPD in 12 Countries. PLOS ONE, 11(4), p.e0152618. |
| 1. Riera, F., Caeiro, J. and Denning, D. (2018). Burden of Serious Fungal Infections in Argentina. *Journal of Fungi*, 4(2), p.51. |
| 1. Caballero, A., Torres-Duque, C., Jaramillo, C., Bolívar, F., Sanabria, F., Osorio, P., Orduz, C., Guevara, D. and Maldonado, D. (2008). Prevalence of COPD in Five Colombian Cities Situated at Low, Medium, and High Altitude (PREPOCOL Study). Chest, 133(2), pp.343-349. |
| 1. Alvarez-Moreno, C., Cortes, J. and Denning, D. (2018). Burden of Fungal Infections in Colombia. Journal of Fungi, 4(2), p.41. |
| 1. Crawford, T., McGrowder, D., Barnett, J., McGaw, B., McKenzie, I. and James, L. (2012). Tobacco-Related Chronic Illnesses: A Public Health Concern for Jamaica. Asian Pacific Journal of Cancer Prevention, 13(9), pp.4733-4738. |
| 1. Zurita, J., Denning, D., Paz-y-Miño, A., Solís, M. and Arias, L. (2017). Serious fungal infections in Ecuador. European Journal of Clinical Microbiology & Infectious Diseases, 36(6), pp.975-981. |
| 1. Jaganath, D., Miranda, J., Gilman, R., Wise, R., Diette, G., Miele, C., Bernabe-Ortiz, A. and Checkley, W. (2015). Prevalence of chronic obstructive pulmonary disease and variation in risk factors across four geographically diverse resource-limited settings in Peru. Respiratory Research, 16(1). |
| 1. Menezes, A., Perez-Padilla, R., Jardim, J., Muiño, A., Lopez, M., Valdivia, G., de Oca, M., Talamo, C., Hallal, P. and Victora, C. (2005). Chronic obstructive pulmonary disease in five Latin American cities (the PLATINO study): a prevalence study. The Lancet, 366(9500), pp.1875-1881. |
| 1. Zhussupov, B., Sharman, A. and Sharman, D. (2018). COPD, metabolic syndrome, respiratory symptoms, and functional incapacity in smokers, ex-smokers, and never-smokers aged 40-59 in Almaty, Kazakhstan: a cross-sectional study. *F1000Research*, 7, p.688. |
| 1. Brakema, E., Tabyshova, A., Kasteleyn, M., Molendijk, E., van der Kleij, R., van Boven, J., Emilov, B., Akmatalieva, M., Mademilov, M., Numans, M., Williams, S., Sooronbaev, T. and Chavannes, N. (2019). High COPD prevalence at high altitude: does household air pollution play a role?. *European Respiratory Journal*, 53(2), p.1801193. |
| 1. Adeloye, D., Chua, S., Lee, C., Basquill, C., Papana, A., Theodoratou, E., Nair, H., Gasevic, D., Sridhar, D., Campbell, H., Chan, K., Sheikh, A. and Rudan, I. (2015). Global and regional estimates of COPD prevalence: Systematic review and meta–analysis. *Journal of Global Health*, 5(2). |
| 1. Zhu, B., Wang, Y., Ming, J., Chen, W. and Zhang, L. (2018). Disease burden of COPD in China: a systematic review. International Journal of Chronic Obstructive Pulmonary Disease, Volume 13, pp.1353-1364. |
| 1. Fang, L., Gao, P., Bao, H., Tang, X., Wang, B., Feng, Y., Cong, S., Juan, J., Fan, J., Lu, K., Wang, N., Hu, Y. and Wang, L. (2018). Chronic obstructive pulmonary disease in China: a nationwide prevalence study. *Lancet Respiratory Medicine*, 6, pp.421-430. |
| 1. Wang, C., Xu, J., Yang, L., Xu, Y. and Zhang, X. *et al* (2018). Prevalence and risk factors of chronic obstructive pulmonary disease in China (the China Pulmonary Health [CPH] study): a national cross-sectional study. *Lancet Respiratory Medicine*. [online] Available at: http://dx.doi.org/10.1016/ S0140-6736(18)30859-6 [Accessed 6 Oct. 2019]. |
| 1. Fukuchi, Y., Nishimura, M., Ichinose, M., Adachi, M., Nagai, A., Kuriyama, T., Takahashi, K., Nishimura, K., Ishioka, S., Aizawa, H. and Zaher, C. (2004). COPD in Japan: the Nippon COPD Epidemiology study. Respirology, 9(4), pp.458-465. |
| 1. Hwang, Y., Park, Y. and Yoo, K. (2017). Recent Trends in the Prevalence of Chronic Obstructive Pulmonary Disease in Korea. Tuberculosis and Respiratory Diseases, 80(3), pp.226-229. |
| 1. Lâm, H., Ekerljung, L., Tu·ò·ng, N., Rönmark, E., Larsson, K. and Lundbäck, B. (2014). Prevalence of COPD by Disease Severity in Men and Women in Northern Vietnam. COPD: Journal of Chronic Obstructive Pulmonary Disease, 11(5), pp.575-581. |
| 1. Pothirat, C., Chaiwong, W., Phetsuk, N., Pisalthanapuna, S., Chetsadaphan, N. and Inchai, J. (2015). A comparative study of COPD burden between urban vs rural communities in northern Thailand. International Journal of Chronic Obstructive Pulmonary Disease, p.1035-1042. |
| 1. Arsalan, A., Shad, Z., Sabah, A., Ahmed, F., Malik, A. and Shakeel, O. (2014). Prevalence and Therapy of Chronic Obstructive Pulmonary Disease in Karachi. *International Journal of Pharmacy Teaching & Practices*, 5(1), pp.867-904. |
| 1. Alam, D., Chowdhury, M., Siddiquee, A., Ahmed, S. and Clemens1, J. (2015). Prevalence and Determinants of Chronic Obstructive Pulmonary Disease (COPD) in Bangladesh. COPD: Journal of Chronic Obstructive Pulmonary Disease, 12, pp.658 – 667. |
| 1. McKay, A., Mahesh, P., Fordham, J. and Majeed, A. (2012). Prevalence of COPD in India: a systematic review. Primary Care Respiratory Journal, 21(3), pp.313-321. |
| 1. Masjedi, M., Sharifi, H., Emami, H., Ghanei, M., Eslaminejad, A., Radmand, G. and Buist, S. (2015). Burden of obstructive lung disease study in Tehran: Prevalence and risk factors of chronic obstructive pulmonary disease. Lung India, 32(6), p.572-577. |
| 1. Al Ghobain, M., Alhamad, E., Alorainy, H., Al Kassimi, F., Lababidi, H. and Al-Hajjaj, M. (2015). The prevalence of chronic obstructive pulmonary disease in Riyadh, Saudi Arabia: a BOLD study. The International Journal of Tuberculosis and Lung Disease, 19(10), pp.1252-1257. |
| 1. Zachariades, A., Zachariadou, T., Adamide, T., Anagnostopoulou, U., Georgiou, A. and Gourgoulianis, K. (2012). Prevalence of Chronic Obstructive Pulmonary Disease in Cyprus: A Population-Based Study. COPD: Journal of Chronic Obstructive Pulmonary Disease, 9(3), pp.259-267. |
| 1. Al Lami, F. and Salim, Z. (2017). Prevalence and determinants of chronic obstructive pulmonary disease among a sample of adult smokers in Baghdad, Iraq, 2014. Eastern Mediterranean Health Journal, 23(2), pp.67-72. |
| 1. Mihaltan, F., Nemes, R., Damarus, I., Farcasanu, D. and Paunescu, B. (2012). Prevalence of Chronic Obstructive Pulmonary Disease (COPD) in Romania. *Chest*, 142(4). |
| 1. Andreeva, E., Pokhaznikova, M., Lebedev, A., Moiseeva, I., Kutznetsova, O. and Degryse, J. (2016). The Prevalence of Chronic Obstructive Pulmonary Disease by the Global Lung Initiative Equations in North-Western Russia. Respiration, 91(1), pp.43-55. |
| 1. Maio, S., Sherrill, D., MacNee, W., Lange, P., Costabel, U., Dahlén, S., Sybrecht, G., Burghuber, O., Stevenson, R., Tønnesen, P., Haeussinger, K., Hedlin, G., Bauer, T., Riedler, J., Nicod, L., Carlsen, K. and Viegi, G. (2012). The European Respiratory Society spirometry tent: a unique form of screening for airway obstruction. European Respiratory Journal, 39(6), pp.1458-1467. |
| 1. Fabricius, P., Løkke, A., Marott, J., Vestbo, J. and Lange, P. (2011). Prevalence of COPD in Copenhagen. Respiratory Medicine, 105(3), pp.410-417. |
| 1. Kainu, A., Rouhos, A., Sovijärvi, A., Lindqvist, A., Sarna, S. and Lundbäck, B. (2013). COPD in Helsinki, Finland: socioeconomic status based on occupation has an important impact on prevalence. Scandinavian Journal of Public Health, 41(6), pp.570-578. |
| 1. Isoaho, R., Puolijoki, H., Huhti, E., Kivelä, S., Laippala, P. and Tala, E. (1994). Prevalence of chronic obstructive pulmonary disease in elderly Finns. Respiratory Medicine, 88(8), pp.571-580. |
| 1. Kanervisto, M., Vasankari, T., Laitinen, T., Heliövaara, M., Jousilahti, P. and Saarelainen, S. (2011). Low socioeconomic status is associated with chronic obstructive airway diseases. Respiratory Medicine, 105(8), pp.1140-1146. |
| 1. Jordan, R., Miller, M., Lam, K., Cheng, K., Marsh, J. and Adab, P. (2012). Sex, susceptibility to smoking and chronic obstructive pulmonary disease: the effect of different diagnostic criteria. Analysis of the Health Survey for England. Thorax, 67(7), pp.600-605. |
| 1. Lindberg, A., Bjerg-Bäcklund, A., Rönmark, E., Larsson, L. and Lundbäck, B. (2006). Prevalence and underdiagnosis of COPD by disease severity and the attributable fraction of smoking. *Respiratory Medicine*, 100(2), pp.264-272. |
| 1. Melville, A., Pless-Mulloli, T., Afolabi, O. and Stenton, S. (2010). COPD prevalence and its association with occupational exposures in a general population. European Respiratory Journal, 36(3), pp.488-493. |
| 1. Blanco, I., Diego, I., Bueno, P., Fernandez, E., Casas-Maldonado, F., Esquinas, C., Soriano, J. and Miravitlles, M. (2017). Geographical distribution of COPD prevalence in Europe, estimated by an inverse distance weighting interpolation technique. International Journal of Chronic Obstructive Pulmonary Disease, Volume 13, pp.57-67. |
| 1. Minas, M., Hatzoglou, C., Karetsi, E., Papaioannou, A., Rita Tsarouchaa, K., Gogou, E., Gourgoulianis, K. and Kostikas, K. (2010). COPD prevalence and the differences between newly and previously diagnosed COPD patients in a spirometry program. Primary Care Respiratory Journal, 19(4), pp.363-370. |
| 1. Viegi, G., Pedreschi, M., Pistelli, F., Di Pede, F., Baldacci, S., Carrozzi, L. and Giuntini, C. (2000). Prevalence of Airways Obstruction in a General Population. Chest, 117(5), pp.339S-345S. |
| 1. Guerriero, M., Caminati, M., Viegi, G., Senna, G., Cesana, G. and Pomari, C. (2015). COPD prevalence in a north-eastern Italian general population. Respiratory Medicine, 109(8), pp.1040-1047. |
| 1. Bárbara, C., Rodrigues, F., Dias, H., Cardoso, J., Almeida, J., Matos, M., Simão, P., Santos, M., Ferreira, J., Gaspar, M., Gnatiuc, L. and Burney, P. (2013). Chronic obstructive pulmonary disease prevalence in Lisbon, Portugal: The burden of obstructive lung disease study. Revista Portuguesa de Pneumologia (English Edition), 19(3), pp.96-105. |
| 1. Arsenijević, V. and Denning, D. (2018). Estimated Burden of Serious Fungal Diseases in Serbia. Journal of Fungi, 4(3), p.76. |
| 1. Miravitlles, M., Murio, c., Tirado-Conde, G., Levy, G. and Muellerova, H. (2008). Geographic differences in clinical characteristics and management of COPD: the EPOCA study. International Journal of Chronic Obstructive Pulmonary Disease, Volume 3, pp.803-814. |
| 1. Cabrera López, C., Juliá Serdá, G., Cabrera Lacalzada, C. and Martín Medina, A. (2014). Prevalence of Chronic Obstructive Pulmonary Disease in the Canary Islands. Archivos de Bronconeumología (English Edition), 50(7), pp.272-277. |
| 1. Vanfleteren, L., Franssen, F., Wesseling, G. and Wouters, E. (2012). The prevalence of chronic obstructive pulmonary disease in Maastricht, the Netherlands. Respiratory Medicine, 106(6), pp.871-874. |
| 1. Roche, N., Dalmay, F., Perez, T., Kuntz, C., Vergnenegre, A., Neukirch, F., Giordanella, J. and Huchon, G. (2008). Impact of chronic airflow obstruction in a working population. European Respiratory Journal, 31(6), pp.1227-1233. |
| 1. Bridevaux, P., Probst-Hensch, N., Schindler, C., Curjuric, I., Felber Dietrich, D., Braendli, O., Brutsche, M., Burdet, L., Frey, M., Gerbase, M., Ackermann-Liebrich, U., Pons, M., Tschopp, J., Rochat, T. and Russi, E. (2010). Prevalence of airflow obstruction in smokers and never-smokers in Switzerland. European Respiratory Journal, 36(6), pp.1259-1269. |
| 1. Shirtcliffe, P., Weatherall, M., Marsh, S., Travers, J., Hansell, A., McNaughton, A., Aldington, S., Muellerova, H. and Beasley, R. (2007). COPD prevalence in a random population survey: a matter of definition. European Respiratory Journal, 30(2), pp.232-239. |
